# Supplementary material for: How long should the fully hillside-closed forest protection be implemented on the Loess Plateau, Shaanxi, China?
Source: PeerJ. 2017 Sep 4;5:e3764. doi: 10.7717/peerj.3764 (PMC5588796; doi:10.7717/peerj.3764)
Supplement: Supplemental Information 1 [file peerj-05-3764-s001.pdf]

Investigation form

Site: Shibu forest farm **Forest type:** *Pinus tabulaeformis* plantation

Protected age: 16 a **Plot area:** 20 m ×20 m **Canopy density**: 0.30

Altitude: 1170 m **Direction:** Southeast **Gradient:** 27° **Position:** Upper

Plot No. : 1 **Investigator:** Lin Hou **Date:** 2004.08.05

No.	Tree species	DBH/ground diameter（cm）	H (m)	Remark
1	*Pinus tabulaeformis*	5.9	1.9	
2	*Pinus tabulaeformis*	6.0	2.0	
3	*Pinus tabulaeformis*	5.2	1.9	
4	*Pinus tabulaeformis*	6.0	2.0	
5	*Pinus tabulaeformis*	12.0	26.0	
6	*Pinus tabulaeformis*	6.0	2.0	
7	*Pinus tabulaeformis*	6.0	1.9	
8	*Pinus tabulaeformis*	5.8	1.9	
9	*Pinus tabulaeformis*	11.0	24.0	
10	*Syringa oblata*	3.4	2.8	
11	*Pinus tabulaeformis*	11.5	21.3	
12	*Pinus tabulaeformis*	5.9	1.8	
13	*Pinus tabulaeformis*	6.0	1.8	
14	*Pinus tabulaeformis*	5.9	1.8	
15	*Pinus tabulaeformis*	12.0	22.0	
16	*Pinus tabulaeformis*	5.9	1.8	
17	*Pinus tabulaeformis*	6.0	1.8	
18	*Pinus tabulaeformis*	6.0	1.9	
19	*Pinus tabulaeformis*	11.4	23.0	
20	*Populus davidiana*	3.6	4.4	
21	*Pinus tabulaeformis*	6.0	2.0	
22	*Pinus tabulaeformis*	12.0	22.0	
23	*Pinus tabulaeformis*	6.0	2.0	
24	*Pinus tabulaeformis*	6.0	1.9	
25	*Pinus tabulaeformis*	12.0	24.0	
26	*Pinus tabulaeformis*	5.9	1.9	
27	*Pinus tabulaeformis*	5.8	2.0	
28	*Pinus tabulaeformis*	5.4	1.9	
29	*Pinus tabulaeformis*	5.7	2.0	

30	*Pinus tabulaeformis*	6.0	2.0	
31	*Pinus tabulaeformis*	12.0	24.0	
32	*Pinus tabulaeformis*	5.8	2.0	
33	*Pinus tabulaeformis*	6.0	1.9	
34	*Pinus tabulaeformis*	6.0	1.8	
35	*Pinus tabulaeformis*	5.8	1.9	
36	*Pinus tabulaeformis*	6.0	1.5	
37	*Pinus tabulaeformis*	6.0	1.8	
38	*Pinus tabulaeformis*	5.4	1.6	
39	*Pinus tabulaeformis*	5.8	1.7	
40	*Pinus tabulaeformis*	6.0	2.0	
41	*Pinus tabulaeformis*	5.4	2.0	
42	*Pinus tabulaeformis*	5.8	1.9	
43	*Pinus tabulaeformis*	6.0	1.6	
44	*Pinus tabulaeformis*	5.6	1.8	
45	*Pinus tabulaeformis*	6.0	2.0	
46	*Pinus tabulaeformis*	5.8	2.0	
47	*Pinus tabulaeformis*	6.0	1.6	
48	*Pinus tabulaeformis*	6.0	2.0	
49	*Pinus tabulaeformis*	5.8	1.6	

Site: Shibu forest farm **Forest type:** *Pinus tabulaeformis* plantation

Protected age: 16 a **Subplot area:** 2 m ×2 m **Mean coverage**: 38%

Investigator: Lin Hou **Date:** 2004.08.06

**Subplot No.**	**Shrub species**	**Individuals**	**Mean height (cm)**	**Coverage (%)**
**1**	*Lespedeza floribunda*	3	45	30
	Rosa hugonis	1	60	5
**2**	*Lespedeza floribunda*	3	50	30
	Sophora viciifolia	1	180	20
**3**	*Lespedeza floribunda*	2	50	25
	Lespedeza dahurica	1	55	10
**4**	*Lespedeza floribunda*	3	50	30
**5**	*Lespedeza dahurica*	1	45	10
	Lespedeza floribunda	3	60	25

Site: Shibu forest farm **Forest type:** *Pinus tabulaeformis* plantation

Protected age: 16 a **Subplot area:** 1 m ×1 m **Mean coverage**: 33%

Investigator: Lin Hou **Date:** 2004.08.06

Subplot No.	Herb species	Individuals	Mean height (cm)	Coverage (%)
**1**	*Bothriochloa ischaemum*	300(branches)	40	30
	Artemisia mongolica	2	100	5
	Artemisia giraldii	1	70	3
**2**	*Bothriochloa ischaemum*	300(branches)	40	30
	Artemisia mongolica	1	70	3
	Patrinia heterophylla	1	30	1
**3**	*Bothriochloa ischaemum*	300(branches)	40	30
	Artemisia mongolica	1	70	3
	Lamium barbatum	1	40	1
**4**	*Bothriochloa ischaemum*	500(branches)	40	35
	Aster tataricus	1	45	2
**5**	*Artemisia mongolica*	4	80	20

Site: Shibu forest farm **Forest type:** *Pinus tabulaeformis* plantation

Protected age: 16 a **Plot area:** 20 m ×20 m **Canopy density**: 0.30

Altitude: 1150 m **Direction:** Southeast **Gradient:** 29° **Position:** Middle

Plot No. : 2 **Investigator:** Lin Hou **Date:** 2004.08.07

No.	Tree species	DBH/ground diameter（cm）	H (m)	Remark
1	*Pinus tabulaeformis*	5.4	1.8	
2	*Populus davidiana*	2.8	2.0	
3	*Pinus tabulaeformis*	6.0	1.9	
4	*Pinus tabulaeformis*	5.4	1.8	
5	*Pinus tabulaeformis*	6.0	2.0	
6	*Pinus tabulaeformis*	12.0	16.0	
7	*Pinus tabulaeformis*	6.0	1.8	
8	*Pinus tabulaeformis*	6.0	1.9	
9	*Syringa oblata*	5.4	2.0	
10	*Pinus tabulaeformis*	5.8	2.0	
11	*Pinus tabulaeformis*	11.8	19.6	
12	*Pinus tabulaeformis*	12.0	18.0	

13	*Pinus tabulaeformis*	5.8	1.7	
14	*Pinus tabulaeformis*	6.0	1.8	
15	*Pinus tabulaeformis*	6.0	1.8	
16	*Pinus tabulaeformis*	12.0	21.2	
17	*Populus davidiana*	1.6	2.0	
18	*Pinus tabulaeformis*	6.0	1.8	
19	*Pinus tabulaeformis*	5.8	1.8	
20	*Pinus tabulaeformis*	6.0	1.9	
21	*Pinus tabulaeformis*	12.0	18.0	
22	*Pinus tabulaeformis*	6.0	1.6	
23	*Pinus tabulaeformis*	12.0	20.0	
24	*Pinus tabulaeformis*	6.0	2.0	
25	*Syringa oblata*	1.8	3.0	
26	*Pinus tabulaeformis*	5.9	1.9	
27	*Pinus tabulaeformis*	11.8	18.6	
28	*Pinus tabulaeformis*	6.0	1.5	
29	*Pinus tabulaeformis*	6.0	1.8	
30	*Pinus tabulaeformis*	5.8	1.9	
31	*Syringa oblata*	4.8	2.0	
32	*Populus davidiana*	2.0	4.0	
33	*Pinus tabulaeformis*	5.8	2.0	
34	*Pinus tabulaeformis*	5.8	2.0	
35	*Pinus tabulaeformis*	12.0	18.8	
36	*Pinus tabulaeformis*	5.80	2.0	
37	*Pinus tabulaeformis*	6.0	1.9	
38	*Pinus tabulaeformis*	6.6	1.8	
39	*Pinus tabulaeformis*	5.8	1.9	
40	*Pinus tabulaeformis*	5.4	2.0	
41	*Pinus tabulaeformis*	12.0	18.0	
42	*Pinus tabulaeformis*	5.0	1.8	
43	*Pinus tabulaeformis*	5.8	1.8	
44	*Pinus tabulaeformis*	6.0	2.0	
45	*Pinus tabulaeformis*	6.0	1.8	
46	*Pinus tabulaeformis*	6.0	2.0	
47	*Pinus tabulaeformis*	6.0	2.0	
48	*Pinus tabulaeformis*	6.0	1.8	

Site: Shibu forest farm **Forest type:** *Pinus tabulaeformis* plantation

Protected age: 16 a **Subplot area:** 1 m ×1 m **Mean coverage**: 37%

Investigator: Lin Hou **Date:** 2004.08.08

Subplot No.	Shrub species	Individuals	Mean height (cm)	Coverage (%)
1	*Rosa hugonis*	1	65	2
	Lespedeza floribunda	3	55	30
	Lespedeza dahurica	1	50	5
2	*Sophora viciifolia*	1	45	1
	Lespedeza floribunda	4	50	30
	Lespedeza dahurica	1	50	5
3	*Lespedeza dahurica*	6	40	40
4	*Lespedeza dahurica*	1	50	10
	Lespedeza floribunda	3	60	30
5	*Lespedeza floribunda*	3	55	20
	Lespedeza dahurica	2	40	10

Site: Shibu forest farm **Forest type:** *Pinus tabulaeformis* plantation

Protected age: 16 a **Subplot area:** 1 m ×1 m **Mean coverage**: 35%

Investigator: Lin Hou **Date:** 2004.08.08

Subplot No.	Herb species	Individuals	Mean height (cm)	Coverage (%)
1	*Bothriochloa ischaemum*	300(branches)	40	30
	Artemisia mongolica	2	70	5
2	*Bothriochloa ischaemum*	300(branches)	40	30
	Artemisia mongolica	1	60	3
	Kengia serotina	1	50	1
3	*Bothriochloa ischaemum*	300(branches)	40	30
	Bupleurum chinense	1	90	2
	Artemisia mongolica	2	70	5
4	*Bothriochloa ischaemum*	300(branches)	40	30
	Artemisia mongolica	3	70	5
5	*Bothriochloa ischaemum*	300(branches)	40	30
	Artemisia mongolica	1	70	2

Site: Shibu forest farm **Forest type:** *Pinus tabulaeformis* plantation

Protected age: 16 a **Plot area:** 20 m ×20 m **Canopy density**: 0.30

Altitude: 1165 m **Direction:** Northeast **Gradient:** 24° **Position:** Middle

Plot No. : 3 **Investigator:** Lin Hou **Date:** 2004.08.09

No.	Tree species	DBH/ground diameter（cm）	H (m)	Remark
1	*Pinus tabulaeformis*	6.0	1.9	
2	*Populus davidiana*	6.0	1.8	
3	*Pinus tabulaeformis*	6.0	2.0	
4	*Syringa oblata*	2.8	3.2	
5	*Pinus tabulaeformis*	5.8	2.0	
6	*Pinus tabulaeformis*	6.0	2.0	
7	*Pinus tabulaeformis*	10.4	18.8	
8	*Pinus tabulaeformis*	5.8	2.0	
9	*Pinus tabulaeformis*	5.6	1.8	
10	*Syringa oblata*	6.0	4.2	
11	*Populus davidiana*	1.8	2.4	
12	*Pinus tabulaeformis*	11.6	18.8	
13	*Pinus tabulaeformis*	5.8	2.0	
14	*Pinus tabulaeformis*	5.8	2.0	
15	*Syringa oblata*	3.6	4.0	
16	*Populus davidiana*	2.4	3.2	
17	*Pinus tabulaeformis*	6.0	2.0	
18	*Pinus tabulaeformis*	12.0	23.0	
19	*Pinus tabulaeformis*	5.4	2.0	
20	*Pinus tabulaeformis*	5.6	2.0	
21	*Populus davidiana*	5.0	1.8	
22	*Pinus tabulaeformis*	5.8	1.6	
23	*Pinus tabulaeformis*	6.0	1.9	
24	*Pinus tabulaeformis*	12.0	22.5	
25	*Pinus tabulaeformis*	5.8	2.0	
26	*Pinus tabulaeformis*	5.4	1.7	
27	*Pinus tabulaeformis*	5.4	1.9	
28	*Pinus tabulaeformis*	10.6	17.8	
29	*Syringa oblata*	5.2	2.0	
30	*Pinus tabulaeformis*	5.4	1.8	
31	*Pinus tabulaeformis*	5.6	1.8	
32	*Pinus tabulaeformis*	5.8	1.9	
33	*Pinus tabulaeformis*	5.8	1.7	
34	*Pinus tabulaeformis*	5.8	2.0	

35	*Syringa oblata*	11.8	22.0	
36	*Populus davidiana*	6.0	1.9	
37	*Pinus tabulaeformis*	6.0	2.0	
38	*Populus davidiana*	3.2	4.6	
39	*Pinus tabulaeformis*	10.4	19.0	
40	*Pinus tabulaeformis*	6.0	1.9	
41	*Pinus tabulaeformis*	5.20	1.7	
42	*Pinus tabulaeformis*	12.0	23.5	
43	*Pinus tabulaeformis*	5.6	1.9	
44	*Pinus tabulaeformis*	5.2	1.6	
45	*Pinus tabulaeformis*	6.0	2.0	
46	*Pinus tabulaeformis*	6.0	2.0	
47	*Pinus tabulaeformis*	5.8	1.7	
48	*Pinus tabulaeformis*	12.0	22.6	
49	*Pinus tabulaeformis*	6.0	1.7	
50	*Pinus tabulaeformis*	6.0	1.8	
51	*Pinus tabulaeformis*	6.0	2.0	
52	*Pinus tabulaeformis*	12.0	22.4	
53	*Pinus tabulaeformis*	6.0	1.7	
54	*Pinus tabulaeformis*	12.0	24.0	
55	*Pinus tabulaeformis*	6.0	2.0	

Site: Shibu forest farm **Forest type:** *Pinus tabulaeformis* plantation

Protected age: 16 a **Subplot area:** 1 m ×1 m **Mean coverage**: 38%

Investigator: Lin Hou **Date:** 2004.08.10

Subplot No.	Shrub species	Individuals	Mean height (cm)	Coverage (%)
1	*Lespedeza floribunda*	2	50	20
	Sophora viciifolia	1	30	2
	Lespedeza dahurica	1	40	10
2	*Lespedeza dahurica*	2	50	15
	Lespedeza floribunda	3	55	20
3	*Rosa hugonis*	1	60	5
	Lespedeza floribunda	4	55	30
	Lespedeza dahurica	1	50	10
4	*Lespedeza floribunda*	3	40	30
	Lespedeza dahurica	1	40	10
5	*Lespedeza dahurica*	2	45	20
	Lespedeza floribunda	2	40	20

Site: Shibu forest farm **Forest type:** *Pinus tabulaeformis* plantation

Protected age: 16 a **Subplot area:** 1 m ×1 m **Mean coverage**: 32%

Investigator: Lin Hou **Date:** 2004.08.10

Subplot No.	Herb species	Individuals	Mean height (cm)	Coverage (%)
**1**	*Bothriochloa ischaemum*	300(branches)	40	30
	Artemisia mongolica	1	70	2
	Astragalus kifonsanicus	1	35	0.5
**2**	*Bothriochloa ischaemum*	300(branches)	40	30
	Artemisia mongolica	1	70	2
**3**	*Bothriochloa ischaemum*	300(branches)	40	30
	Artemisia mongolica	1	70	1
**4**	*Bothriochloa ischaemum*	300(branches)	40	30
	Artemisia mongolica	1	70	1
**5**	*Bothriochloa ischaemum*	300(branches)	40	30
	Artemisia mongolica	1	70	1
	Viola chaerophylloides	1	15	0.5

Site: Shibu forest farm **Forest type:** *Pinus tabulaeformis* plantation

Protected age: 16 a **Plot area:** 20 m ×20 m **Canopy density**: 0.30

Altitude: 1135 m **Direction:** Northeast **Gradient:** 26° **Position:** Lower

Plot No. : 4 **Investigator:** Lin Hou **Date:** 2004.08.11

No.	Tree species	DBH/ground diameter（cm）	H (m)	Remark
1	*Pinus tabulaeformis*	6.0	2.0	
2	*Pinus tabulaeformis*	12.0	23.8	
3	*Pinus tabulaeformis*	6.0	1.8	
4	*Pinus tabulaeformis*	6.0	2.0	
5	*Pinus tabulaeformis*	12.0	23.4	
6	*Pinus tabulaeformis*	6.0	2.0	
7	*Pinus tabulaeformis*	6.0	1.8	
8	*Pinus tabulaeformis*	6.0	2.0	
9	*Populus davidiana*	6.0	3.2	
10	*Pinus tabulaeformis*	12.0	22.8	
11	*Pinus tabulaeformis*	6.0	1.8	
12	*Pinus tabulaeformis*	5.6	1.9	

13	*Pinus tabulaeformis*	6.0	2.0	
14	*Pinus tabulaeformis*	12.0	21.8	
15	*Pinus tabulaeformis*	6.0	2.0	
16	*Pinus tabulaeformis*	6.0	2.0	
17	*Pinus tabulaeformis*	5.8	1.8	
18	*Pinus tabulaeformis*	12.0	22.0	
19	*Pinus tabulaeformis*	6.0	1.8	
20	*Pinus tabulaeformis*	6.0	2.0	
21	*Pinus tabulaeformis*	6.0	2.0	
22	*Populus davidiana*	4.8	3.4	
23	*Pinus tabulaeformis*	12.0	24.0	
24	*Pinus tabulaeformis*	6.0	1.8	
25	*Pinus tabulaeformis*	6.0	2.0	
26	*Pinus tabulaeformis*	6.0	2.0	
27	*Pinus tabulaeformis*	11.8	16.8	
28	*Pinus tabulaeformis*	12.0	23.0	
29	*Pinus tabulaeformis*	6.0	1.8	
30	*Syringa oblata*	9.6	12.0	
31	*Pinus tabulaeformis*	6.0	1.9	
32	*Pinus tabulaeformis*	12.0	24.2	
33	*Pinus tabulaeformis*	6.0	1.8	
34	*Pinus tabulaeformis*	6.0	1.8	
35	*Pinus tabulaeformis*	6.0	2.0	
36	*Pinus tabulaeformis*	12.0	22.6	
37	*Pinus tabulaeformis*	6.0	1.8	
38	*Pinus tabulaeformis*	12.0	23.8	
39	*Pinus tabulaeformis*	6.0	2.0	
40	*Pinus tabulaeformis*	6.0	2.0	
41	*Pinus tabulaeformis*	6.0	1.9	
42	*Pinus tabulaeformis*	12.0	23.0	
43	*Pinus tabulaeformis*	6.0	1.8	
44	*Pinus tabulaeformis*	6.0	1.9	
45	*Pinus tabulaeformis*	12.0	23.8	
46	*Pinus tabulaeformis*	12.0	24.4	
47	*Pinus tabulaeformis*	6.0	2.0	

Site: Shibu forest farm **Forest type:** *Pinus tabulaeformis* plantation

Protected age: 16 a **Subplot area:** 1 m ×1 m **Mean coverage**: 37%

Investigator: Lin Hou **Date:** 2004.08.12

Subplot No.	Shrub species	Individuals	Mean height (cm)	Coverage (%)
1	*Lespedeza floribunda*	2	50	20
	Lespedeza dahurica	1	45	10
2	*Lespedeza floribunda*	1	45	10
	Lespedeza dahurica	3	50	30
3	*Lespedeza floribunda*	1	40	5
	Lespedeza dahurica	3	45	30
4	*Lespedeza floribunda*	2	50	20
	Lespedeza dahurica	2	55	20
5	*Lespedeza floribunda*	3	50	30
	Lespedeza dahurica	1	40	10

Site: Shibu forest farm **Forest type:** *Pinus tabulaeformis* plantation

Protected age: 16 a **Subplot area:** 1 m ×1 m **Mean coverage**: 35%

Investigator: Lin Hou **Date:** 2004.08.12

Subplot No.	Herb species	Individuals	Mean height (cm)	Coverage (%)
1	*Bothriochloa ischaemum*	300(branches)	40	30
	Artemisia mongolica	4	70	10
2	*Bothriochloa ischaemum*	300(branches)	40	30
	Artemisia mongolica	3	70	8
3	*Bothriochloa ischaemum*	300(branches)	40	30
	Artemisia mongolica	2	70	6
4	*Bothriochloa ischaemum*	300(branches)	40	30
	Artemisia mongolica	1	70	3
5	*Bothriochloa ischaemum*	600(branches)	40	35

Site: forest farm **Forest type:** *Pinus tabulaeformis* plantation

Protected age: 16 a **Plot area:** 20 m ×20 m **Canopy density**: 0.30

Altitude: 1295 m **Direction:** North **Gradient:** 19° **Position:** Middle

Plot No. : 5 **Investigator:** Lin Hou **Date:** 2004.08.13

No.	Tree species	DBH/ground diameter（cm）	H (m)	Remark
1	*Pinus tabulaeformis*	3.2	1.8	
2	*Populus davidiana*	2.0	2.4	
3	*Pinus tabulaeformis*	4.8	1.8	

4	*Pinus tabulaeformis*	4.2	2.0	
5	*Pinus tabulaeformis*	11.8	24.6	
6	*Pinus tabulaeformis*	6.0	2.0	
7	*Pinus tabulaeformis*	11.8	23.8	
8	*Pinus tabulaeformis*	5.2	1.7	
9	*Syringa oblata*	1.5	2.4	
10	*Pinus tabulaeformis*	5.6	2.0	
11	*Pinus tabulaeformis*	5.4	1.8	
12	*Pinus tabulaeformis*	5.6	2.0	
13	*Pinus tabulaeformis*	10.8	15.0	
14	*Pinus tabulaeformis*	9.8	13.5	
15	*Syringa oblata*	2.2	3.6	
16	*Pinus tabulaeformis*	5.2	1.8	
17	*Pinus tabulaeformis*	2.8	1.6	
18	*Pinus tabulaeformis*	6.0	2.0	
19	*Pinus tabulaeformis*	11.8	23.4	
20	*Pinus tabulaeformis*	6.0	2.0	
21	*Pinus tabulaeformis*	3.0	1.8	
22	*Pinus tabulaeformis*	4.6	2.0	
23	*Pinus tabulaeformis*	6.0	2.0	
24	*Pinus tabulaeformis*	11.2	21.6	
25	*Pinus tabulaeformis*	6.0	2.0	
26	*Pinus tabulaeformis*	12.0	21.4	
27	*Pinus tabulaeformis*	4.0	1.6	
28	*Pinus tabulaeformis*	9.8	15.4	
29	*Pinus tabulaeformis*	5.4	1.9	
30	*Pinus tabulaeformis*	5.2	1.8	
31	*Pinus tabulaeformis*	12.0	23.0	
32	*Pinus tabulaeformis*	6.0	2.0	
33	*Pinus tabulaeformis*	4.6	2.0	
34	*Pinus tabulaeformis*	8.8	14.8	
35	*Pinus tabulaeformis*	5.2	2.0	
36	*Pinus tabulaeformis*	3.2	1.8	
37	*Pinus tabulaeformis*	5.8	2.0	
38	*Pinus tabulaeformis*	11.6	23.8	
39	*Pinus tabulaeformis*	2.0	1.6	
40	*Pinus tabulaeformis*	5.0	2.0	
41	*Populus davidiana*	3.2	4.8	
42	*Pinus tabulaeformis*	6.0	2.0	

43	*Pinus tabulaeformis*	10.8	16.8	
44	*Pinus tabulaeformis*	5.4	2.0	
45	*Pinus tabulaeformis*	5.6	2.0	
46	*Pinus tabulaeformis*	4.0	1.6	
47	*Pinus tabulaeformis*	11.4	23.4	
48	*Pinus tabulaeformis*	11.6	20.8	
49	*Pinus tabulaeformis*	12.0	23.6	
50	*Pinus tabulaeformis*	5.6	1.8	
51	*Pinus tabulaeformis*	3.2	1.6	
52	*Pinus tabulaeformis*	5.8	1.8	
53	*Pinus tabulaeformis*	11.8	23.8	
54	*Pinus tabulaeformis*	6.0	2.0	

Site: forest farm **Forest type:** *Pinus tabulaeformis* plantation

Protected age: 16 a **Subplot area:** 1 m ×1 m **Mean coverage**: 31%

Investigator: Lin Hou **Date:** 2004.08.14

**Subplot No.**	**Shrub species**	**Individuals**	**Mean height (cm)**	**Coverage (%)**
**1**	*Lespedeza floribunda*	2	40	20
	Lespedeza dahurica	1	40	10
**2**	*Lespedeza floribunda*	3	40	25
	Lespedeza dahurica	1	30	5
**3**	*Lespedeza floribunda*	1	40	10
	Lespedeza dahurica	3	35	25
**4**	*Lespedeza floribunda*	2	50	20
	Lespedeza dahurica	1	45	10
**5**	*Lespedeza floribunda*	4	50	30

Site: Shibu forest farm **Forest type:** *Pinus tabulaeformis* plantation

Protected age: 16 a **Subplot area:** 1 m ×1 m **Mean coverage**: 31%

Investigator: Lin Hou **Date:** 2004.08.14

**Subplot No.**	**Herb species**	**Individuals**	**Mean height (cm)**	**Coverage (%)**
**1**	*Bothriochloa ischaemum*	400(branches)	40	30
	Rhaponticum uniflorum	1	60	1
**2**	*Bothriochloa ischaemum*	300(branches)	40	30

	Artemisia mongolica	2	70	5
**3**	*Artemisia mongolica*	5	70	15
	Scutellaria baicalensis	1	65	2
**4**	*Bothriochloa ischaemum*	300(branches)	40	25
	Aster tataricus	1	45	2
**5**	*Bothriochloa ischaemum*	300(branches)	40	30
	Artemisia mongolica	1	70	2

Site: Guanzhuang forest farm **Forest type:** *Pinus tabulaeformis* plantation

Protected age: 30 a **Plot area:** 20 m ×20 m **Canopy density**: 0.60

Altitude: 1154 m **Direction:** North **Gradient:** 22.3° **Position:** Middle

Plot No. : 6 **Investigator:** Lin Hou **Date:** 2004.08.15

No.	Tree species	DBH/ground diameter（cm）	H (m)	Remark
1	*Pinus tabulaeformis*	11.8	11.4	
2	*Pinus tabulaeformis*	19.6	15.2	
3	*Pinus tabulaeformis*	11.4	11.0	
4	*Pinus tabulaeformis*	19.4	17.2	
5	*Quercus Liaotungensis*	13.6	15.0	
6	*Pinus tabulaeformis*	11.4	12.8	
7	*Pinus tabulaeformis*	11.2	13.6	
8	*Pinus tabulaeformis*	11.8	14.0	
9	*Pinus tabulaeformis*	5.8	1.8	
10	*Pinus tabulaeformis*	19.6	15.4	
11	*Pinus tabulaeformis*	11.4	11.8	
12	*Pinus tabulaeformis*	10.8	11.0	
13	*Pinus tabulaeformis*	5.9	1.9	
14	*Quercus Liaotungensis*	10.4	12.8	
15	*Pinus tabulaeformis*	11.4	12.9	
16	*Pinus tabulaeformis*	18.9	12.8	
17	*Pinus tabulaeformis*	11.6	14.8	
18	*Pinus tabulaeformis*	5.7	1.8	
19	*Pinus tabulaeformis*	19.8	16.5	
20	*Pinus tabulaeformis*	11.8	12.8	
21	*Pinus tabulaeformis*	11.6	12.0	
22	*Pinus tabulaeformis*	11.9	13.5	
23	*Pinus tabulaeformis*	18.4	14.2	
24	*Prunus tomenosa*	2.6	3	

25	*Pinus tabulaeformis*	19.8	20.4	
26	*Pinus tabulaeformis*	12.0	12.8	
27	*Pinus tabulaeformis*	11.6	12.0	
28	*Pinus tabulaeformis*	11.4	13.0	
29	*Pinus tabulaeformis*	11.6	13.4	
30	*Pinus tabulaeformis*	19.6	18	
31	*Pinus tabulaeformis*	12.0	11.8	
32	*Pinus tabulaeformis*	5.8	2.0	
33	*Pinus tabulaeformis*	18.8	11.4	
34	*Pinus tabulaeformis*	19.6	14.6	
35	*Pinus tabulaeformis*	16.4	13.6	
36	*Pinus tabulaeformis*	18.6	17.0	
37	*Pinus tabulaeformis*	11.9	13.8	
38	*Pinus tabulaeformis*	36.0	22.2	
39	*Pinus tabulaeformis*	1.3	0.2	
40	*Pinus tabulaeformis*	11.8	16.2	
41	*Pinus tabulaeformis*	19.8	24.4	
42	*Pinus tabulaeformis*	18.8	14.6	
43	*Pinus tabulaeformis*	5.4	2.0	
44	*Prunus davidiana*	2.8	4.2	
45	*Pinus tabulaeformis*	19.4	16.0	
46	*Xanthoceras sorbifolia*	3.6	4.5	
47	*Pinus tabulaeformis*	5.2	1.8	

Site: Guanzhuang forest farm **Forest type:** *Pinus tabulaeformis* plantation

Protected age: 30 a **Subplot area:** 2 m ×2 m **Mean coverage**: 25%

Investigator: Lin Hou **Date:** 2004.08.16

**Subplot No.**	**Shrub species**	**Individuals**	**Mean height (cm)**	**Coverage (%)**
**1**	*Lespedeza dahurica*	3	45	25
	Lonicera ferdinandii	2	50	4
	Ostryopsis davidiana	1	150	3
**2**	*Acer ginnala*	2	220	10
	Cotoneaster multiflorus	1	180	2
	Clematis brevicaudata	1	130	2
**3**	*Lespedeza dahurica*	2	40	20

	Ziziphus jujube var.spinosus	1	80	8
**4**	*Acer ginnala*	1	150	7
	Lonicera ferdinandii	3	60	7
**5**	*Lespedeza dahurica*	3	50	30
	Sophora viciifolia	1	220	5

Site: Guanzhuang forest farm **Forest type:** *Pinus tabulaeformis* plantation

Protected age: 30 a **Subplot area:** 1 m ×1 m **Mean coverage**: 28%

Investigator: Lin Hou **Date:** 2004.08.16

Subplot No.	Herb species	Individuals	Mean height (cm)	Coverage (%)
**1**	*Bothriochloa ischaemum*	300(branches)	35	20
	Patrinia heterophylla	1	50	1
	Kengia serotina	3	55	10
**2**	*Artemisia mongolica*	3	80	15
	Bupleurum chinense	5	80	20
**3**	*Viola yedoensis*	5	10	10
	Aster tataricus	8	45	15
**4**	*Bothriochloa ischaemum*	400(branches)	40	25
	Carpesium divaricatum	3	70	10
**5**	*Thalictrum prezewalskii*	4	100	20
	Saussurea salsa	5	30	15

Site: Guanzhuang forest farm **Forest type:** *Pinus tabulaeformis* plantation

Protected age: 30 a **Plot area:** 20 m ×20 m **Canopy density**: 0.50

Altitude: 1167 m **Direction:** Northwest **Gradient:** 24° **Position:** Lower

Plot No. : 7 **Investigator:** Lin Hou **Date:** 2004.08.17

No.	Tree species	DBH/ground diameter（cm）	H (m)	Remark
1	*Pinus tabulaeformis*	1.8	0.3	
2	*Pinus tabulaeformis*	12.0	5.6	
3	*Prunus davidiana*	11.8	12.2	
4	*Pinus tabulaeformis*	19.6	8.4	
5	*Pinus tabulaeformis*	11.6	4.8	

6	*Betula platyphylla*	2.4	3.2	
7	*Pinus tabulaeformis*	12.0	5.2	
8	*Pinus tabulaeformis*	5.8	1.9	
9	*Pinus tabulaeformis*	19.4	9.0	
10	*Pinus tabulaeformis*	11.6	4.9	
11	*Conus walteri wanger*	1.8	2.2	
12	*Pinus tabulaeformis*	19.8	9.2	
13	*Pinus tabulaeformis*	12.0	7.2	
14	*Quercus Liaotungensis*	4.8	1.4	
15	*Pinus tabulaeformis*	19.4	9.0	
16	*Pinus tabulaeformis*	11.8	5.6	
17	*Betula platyphylla*	3.4	4.8	
18	*Pinus tabulaeformis*	20.0	10.8	
19	*Pinus tabulaeformis*	12.0	5.4	
20	*Pinus tabulaeformis*	5.6	1.8	
21	*Quercus Liaotungensis*	3.4	4.0	
22	*Pinus tabulaeformis*	12.0	7.0	
23	*Pinus tabulaeformis*	19.4	9.2	
24	*Betula platyphylla*	3.0	2.8	
25	*Pinus tabulaeformis*	19.2	10.0	
26	*Pinus tabulaeformis*	11.4	7.0	
27	*Syringa oblata*	6.0	3.2	
28	*Pinus tabulaeformis*	19.8	9.4	
29	*Pinus tabulaeformis*	12.0	7.8	
30	*Betula platyphylla*	3.6	3.8	
31	*Pinus tabulaeformis*	48.0	15.0	
32	*Pinus tabulaeformis*	12.0	7.6	
33	*Pinus tabulaeformis*	19.4	9.2	
34	*Pinus tabulaeformis*	20.0	9.6	
35	*Pinus tabulaeformis*	12.0	5.0	
36	*Pinus tabulaeformis*	11.8	4.8	

Site: Guanzhuang forest farm **Forest type:** *Pinus tabulaeformis* plantation

Protected age: 30 a **Subplot area:** 2 m ×2 m **Mean coverage**: 35%

Investigator: Lin Hou **Date:** 2004.08.18

Subplot No.	Shrub species	Individuals	Mean height (cm)	Coverage (%)

1	*Lespedeza dahurica*	3	45	25
	Spiraea fritschiana	2	130	15
	Acer ginnala	2	90	5
	Sophora viciifolia	1	150	3
2	*Lespedeza dahurica*	3	60	25
	Ziziphus jujube var.spinosus	2	100	12
3	*Lespedeza dahurica*	2	40	20
	Clematis fruticosa	1	50	3
4	*Lespedeza dahurica*	2	40	20
	Spiraea fritschiana	1	150	10
5	*Lespedeza dahurica*	3	50	30
	Periploca sepium	1	60	1
	Rhamnus davurica	2	90	2
	Rhamnus utilis	110	1	2

Site: Guanzhuang forest farm **Forest type:** *Pinus tabulaeformis* plantation

Protected age: 30 a **Subplot area:** 1 m ×1 m **Mean coverage**: %

Investigator: Lin Hou **Date:** 2004.08.18

**Subplot No.**	**Herb species**	**Individuals**	**Mean height (cm)**	**Coverage (%)**
1	*Adenophora potaninii*	3	40	10
	Kengia serotina	4	50	15
2	*Bothriochloa ischaemum*	350(branches)	30	20
	Leontopodium leontopodioides	4	35	15
3	*Spodiopogon sibiricus*	3	80	20
	Lilium pumilum	2	50	10
4	*Patrinia heterophylla*	1	60	1
	Lysimachia barystachys	5	80	25
5	*Adenophora stricta*	5	80	20
	Kengia serotina	3	60	15

Site: Guanzhuang forest farm **Forest type:** *Pinus tabulaeformis* plantation

Protected age: 30 a **Plot area:** 20 m ×20 m **Canopy density**: 0.70

Altitude: 1180 m **Direction:** South **Gradient:** 35° **Position:** Upper

Plot No. : 8 **Investigator:** Lin Hou **Date:** 2004.08.19

No.	Tree species	DBH/ground diameter（cm）	H (m)	Remark
1	*Pinus tabulaeformis*	1.6	0.3	
2	*Pinus tabulaeformis*	5.8	1.8	
3	*Pinus tabulaeformis*	18.8	11.4	
4	*Quercus Liaotungensis*	3.6	4.2	
5	*Pinus tabulaeformis*	11.8	8.2	
6	*Pinus tabulaeformis*	19.4	11.6	
7	*Pinus tabulaeformis*	11.6	7.8	
8	*Pinus tabulaeformis*	6.0	1.9	
9	*Pinus tabulaeformis*	17.6	8.4	
10	*Pinus tabulaeformis*	18.6	11.8	
11	*Pinus tabulaeformis*	11.4	8.0	
12	*Syringa oblata*	2.8	3.6	
13	*Pinus tabulaeformis*	19.8	11.0	
14	*Pinus tabulaeformis*	10.8	7.2	
15	*Pinus tabulaeformis*	6.0	2.0	
16	*Pinus tabulaeformis*	17.2	10.4	
17	*Pinus tabulaeformis*	12.0	6.8	
18	*Pinus tabulaeformis*	17.0	8.0	
19	*Pinus tabulaeformis*	10.6	6.9	
20	*Pinus tabulaeformis*	14.5	10.5	
21	*Betula platyphylla*	2.4	3.2	
22	*Pinus tabulaeformis*	13.0	9.6	
23	*Pinus tabulaeformis*	41.0	14.6	
24	*Pinus tabulaeformis*	12.8	6.5	
25	*Pinus tabulaeformis*	12.2	5.3	
26	*Prunus davidiana*	3.0	4.2	
27	*Pinus tabulaeformis*	11.8	8.0	
28	*Pinus tabulaeformis*	13.0	8.2	
29	*Pinus tabulaeformis*	11.6	10.8	
30	*Pinus tabulaeformis*	11.4	8.4	
31	*Pinus tabulaeformis*	13.6	8.6	
32	*Pinus tabulaeformis*	12.0	8.2	
33	*Pinus tabulaeformis*	11.8	11.0	
34	*Prunus tomenosa*	2.8	3.2	

Site: Guanzhuang forest farm **Forest type:** *Pinus tabulaeformis* plantation

Protected age: 30 a **Subplot area:** 2 m ×2 m **Mean coverage**: 29%

Investigator: Lin Hou **Date:** 2004.08.20

Subplot No.	Shrub species	Individuals	Mean height (cm)	Coverage (%)
1	*Acer ginnala*	1	80	5
	Sophora viciifolia	2	70	2
2	*Lespedeza dahurica*	2	60	20
	Berberis dielsiana	1	150	3
3	*Lespedeza dahurica*	1	50	15
	Ziziphus jujube var.spinosus	1	120	15
4	*Lespedeza dahurica*	3	60	30
	Spiraea fritschiana	3	100	20
	Acer ginnala	1	90	5
5	*Lespedeza dahurica*	3	50	30
	Lonicera ferdinandii	1	30	2

Site: Guanzhuang forest farm **Forest type:** *Pinus tabulaeformis* plantation

Protected age: 30 a **Subplot area:** 1 m ×1 m **Mean coverage**: 30%

Investigator: Lin Hou **Date:** 2004.08.20

Subplot No.	Herb species	Individuals	Mean height (cm)	Coverage (%)
1	*Bothriochloa ischaemum*	200(branches)	40	18
	Melissitus ruthenicus	3	85	10
2	*Agrimonia pilosa*	5	10	15
	Kengia serotina	8	50	20
3	*Bothriochloa ischaemum*	500(branches)	45	30
	Pennisetum clandestinum	3	90	10
4	*Artemisia gmelinii*	3	60	5
	Polygonatum odoratum	6	40	30
5	*Discorea nippnica*	4	110	15
	Gentiana macrophylla	2	80	5

Site: Guanzhuang forest farm **Forest type:** *Pinus tabulaeformis* plantation

Protected age: 30 a **Plot area:** 20 m ×20 m **Canopy density**: 0.70

Altitude: 1165 m **Direction:** South **Gradient:** 35° **Position:** Lower

Plot No. : 9 **Investigator:** Lin Hou **Date:** 2004.08.21

No.	Tree species	DBH/ground diameter（cm）	H (m)	Remark
1	*Pinus tabulaeformis*	19.8	21.0	
2	*Pinus tabulaeformis*	11.6	11.4	
3	*Betula platyphylla*	2.8	3.2	
4	*Pinus tabulaeformis*	19.6	19.8	
5	*Pinus tabulaeformis*	11.6	11.5	
6	*Pinus tabulaeformis*	11.8	11.5	
7	*Quercus Liaotungensis*	4.5	5.6	
8	*Pinus tabulaeformis*	1.6	0.3	
9	*Pinus tabulaeformis*	11.8	11.2	
10	*Pinus tabulaeformis*	19.6	18.2	
11	*Pinus tabulaeformis*	11.4	11.0	
12	*Pinus tabulaeformis*	11.6	11.4	
13	*Pinus tabulaeformis*	11.2	11.0	
14	*Prunus tomenosa*	1.8	2.4	
15	*Pinus tabulaeformis*	1.8	0.3	
16	*Pinus tabulaeformis*	11.4	10.8	
17	*Pinus tabulaeformis*	17.8	18.0	
18	*Pinus tabulaeformis*	5.8	2.0	
19	*Pinus tabulaeformis*	11.6	10.6	
20	*Pinus tabulaeformis*	19.8	19.5	
21	*Pinus tabulaeformis*	11.8	10.8	
22	*Pinus tabulaeformis*	19.6	21.4	
23	*Pinus tabulaeformis*	5.4	1.9	
24	*Pinus tabulaeformis*	19.4	20.4	
25	*Syringa oblata*	2.2	2.8	
26	*Pinus tabulaeformis*	11.6	11.5	
27	*Pinus tabulaeformis*	19.4	21.8	
28	*Pinus tabulaeformis*	11.4	11.2	
29	*Pinus tabulaeformis*	5.2	1.8	
30	*Pinus tabulaeformis*	19.6	21.8	
31	*Pinus tabulaeformis*	19.5	20.2	

32	*Pinus tabulaeformis*	88.0	28.6	
33	*Prunus davidiana*	3.2	3.6	
34	*Pinus tabulaeformis*	19.8	21.6	
35	*Pinus tabulaeformis*	19.4	21.6	
36	*Pinus tabulaeformis*	19.8	22.0	
37	*Pinus tabulaeformis*	19.0	19.9	
38	*Pinus tabulaeformis*	11.8	11.6	
39	*Pinus tabulaeformis*	19.6	21.0	
40	*Pinus tabulaeformis*	11.4	11.8	
41	*Pinus tabulaeformis*	11.4	11.6	
42	*Betula platyphylla*	2.8	3.0	
43	*Pinus tabulaeformis*	19.5	22.4	
44	*Pinus tabulaeformis*	19.0	21.8	
45	*Pinus tabulaeformis*	11.8	11.6	
46	*Pinus tabulaeformis*	4.8	1.6	
47	*Pinus tabulaeformis*	11.8	11.2	
48	*Pinus tabulaeformis*	11.8	11.3	
49	*Pinus tabulaeformis*	19.6	20.5	
50	*Pinus tabulaeformis*	19.6	21.6	
51	*Pinus tabulaeformis*	19.6	20.4	
52	*Pinus tabulaeformis*	19.2	21.8	
53	*Pinus tabulaeformis*	19.5	22.4	
54	*Pinus tabulaeformis*	11.2	11.0	
55	*Conus walteri wanger*	2.4	3.0	
56	*Pinus tabulaeformis*	11.6	11.0	
57	*Pinus tabulaeformis*	11.4	11.0	
58	*Pinus tabulaeformis*	5.6	1.8	
59	*Pinus tabulaeformis*	11.2	11.5	
60	*Pinus tabulaeformis*	11.8	11.4	
61	*Pinus tabulaeformis*	11.6	11.2	
62	*Pinus tabulaeformis*	5.4	1.7	

Site: Guanzhuang forest farm **Forest type:** *Pinus tabulaeformis* plantation

Protected age: 30 a **Subplot area:** 2 m ×2 m **Mean coverage**: 36%

Investigator: Lin Hou **Date:** 2004.08.22

Subplot No.	Shrub species	Individuals	Mean height (cm)	Coverage (%)
1	*Lespedeza dahurica*	2	45	20
	Spiraea fritschiana	2	120	20
	Rubus corchorifolius	1	110	1
2	*Lespedeza dahurica*	1	60	25
	Acer ginnala	1	100	7
3	*Lespedeza dahurica*	3	40	20
	Spiraea fritschiana	1	180	15
4	*Lespedeza dahurica*	2	60	25
	Sophora viciifolia	1	120	1
5	*Lespedeza dahurica*	3	50	30
	Spiraea fritschiana	2	90	15
	Sophora viciifolia	2	90	2

Site: Guanzhuang forest farm **Forest type:** *Pinus tabulaeformis* plantation

Protected age: 30 a **Subplot area:** 1 m ×1 m **Mean coverage**: 27%

Investigator: Lin Hou **Date:** 2004.08.22

Subplot No.	Herb species	Individuals	Mean height (cm)	Coverage (%)
1	*Bothriochloa ischaemum*	300(branches)	35	20
	Kengia serotina	4	55	15
2	*Anaphalis margaritacea*	5	40	20
	Potentilla supina	3	35	15
3	*Patrinia heterophylla*	2	30	2
	Scutellaria baicalensis	5	80	20
4	*Adenophora stricta*	6	70	20
	Saussurea nivea	5	40	15
5	*Artemisia mongolica*	2	70	10

Site: Guanzhuang forest farm **Forest type:** *Pinus tabulaeformis* plantation

Protected age: 30 a **Plot area:** 20 m ×20 m **Canopy density**: 0.60

Altitude: 1180 m **Direction:** South **Gradient:** 22° **Position:** Lower

Plot No. : 10 **Investigator:** Lin Hou **Date:** 2004.08.23

No.	Tree species	DBH/ground diameter（cm）	H (m)	Remark
1	*Pinus tabulaeformis*	1.8	0.3	
2	*Pinus tabulaeformis*	5.6	1.8	
3	*Pinus tabulaeformis*	5.4	1.8	
4	*Quercus Liaotungensis*	3.2	4.6	
5	*Pinus tabulaeformis*	11.8	12.6	
6	*Pinus tabulaeformis*	10.4	12.0	
7	*Betula platyphylla*	2.8	4.0	
8	*Pinus tabulaeformis*	11.5	12.2	
9	*Pinus tabulaeformis*	19.6	20.4	
10	*Pinus tabulaeformis*	11.8	12.4	
11	*Pinus tabulaeformis*	19.8	21.0	
12	*Pinus tabulaeformis*	11.9	12.8	
13	*Pinus tabulaeformis*	19.4	20.0	
14	*Pinus tabulaeformis*	11.2	12.6	
15	*Pinus tabulaeformis*	5.2	1.8	
16	*Pinus tabulaeformis*	5.0	1.6	
17	*Pinus tabulaeformis*	48.0	23.4	
18	*Pinus tabulaeformis*	1.6	0.3	
19	*Pinus tabulaeformis*	11.8	12.8	
20	*Pinus tabulaeformis*	18.8	19.6	
21	*Pinus tabulaeformis*	11.6	12.5	
22	*Pinus tabulaeformis*	19.2	19.8	
23	*Pinus tabulaeformis*	11.8	12.8	
24	*Quercus Liaotungensis*	3.5	4.8	
25	*Pinus tabulaeformis*	5.6	1.8	
26	*Pinus tabulaeformis*	11.4	12.5	
27	*Pinus tabulaeformis*			
28	*Pinus tabulaeformis*	11.6	12.4	
29	*Prunus davidiana*	3.2	4.4	
30	*Pinus tabulaeformis*	11.8	12.6	
31	*Pinus tabulaeformis*	19	20.4	
32	*Pinus tabulaeformis*	11.4	12.6	
33	*Pinus tabulaeformis*	19.2	20.5	
34	*Pinus tabulaeformis*	11.2	12.4	
35	*Pinus tabulaeformis*	5.0	1.6	
36	*Pinus tabulaeformis*	19.6	21.0	
37	*Pinus tabulaeformis*	5.5	1.8	
38	*Pinus tabulaeformis*	11.2	12.6	

39	*Pinus tabulaeformis*	19.8	22.0	
40	*Pinus tabulaeformis*	11.6	12.8	
41	*Pinus tabulaeformis*	11.4	12.8	
42	*Prunus tomenosa*	3.0	4.2	
43	*Pinus tabulaeformis*	11.6	12.6	
44	*Pinus tabulaeformis*	19.4	20.6	
45	*Pinus tabulaeformis*	11.2	12.4	
46	*Pinus tabulaeformis*	19.4	20.0	
47	*Pinus tabulaeformis*	18.5	19.0	
48	*Pinus tabulaeformis*	11.8	13.0	
49	*Pinus tabulaeformis*	18.8	19.4	
50	*Pinus tabulaeformis*	19	19.5	
51	*Pinus tabulaeformis*	11.8	13.2	
52	*Pinus tabulaeformis*	18	19.0	
53	*Pinus tabulaeformis*	18.2	19.2	
54	*Pinus tabulaeformis*	11.6	12.8	
55	*Pinus tabulaeformis*	17.5	19	
56	*Syringa oblata*	2.8	3.8	
57	*Pinus tabulaeformis*	12.8	13.6	
58	*Pinus tabulaeformis*	15.8	17	
59	*Pinus tabulaeformis*	11.2	12.6	
60	*Pinus tabulaeformis*	13.8	15	
61	*Pinus tabulaeformis*	14.6	15.4	
62	*Pinus tabulaeformis*	11.4	12.4	
63	*Betula platyphylla*	3.6	5	
64	*Pinus tabulaeformis*	13.2	13.8	

Site: Guanzhuang forest farm **Forest type:** *Pinus tabulaeformis* plantation

Protected age: 30 a **Subplot area:** 2 m ×2 m **Mean coverage**: 28%

Investigator: Lin Hou **Date:** 2004.08.24

**Subplot No.**	**Shrub species**	**Individuals**	**Mean height (cm)**	**Coverage (%)**
**1**	*Lespedeza dahurica*	3	45	25
	Acer ginnala	2	75	3
**2**	*Lespedeza dahurica*	2	60	25
	Sophora viciifolia	1	110	2
**3**	*Lespedeza dahurica*	1	60	20

	Spiraea fritschiana	1	90	10
**4**	*Lespedeza dahurica*	3	40	25
	Lonicera ferdinandii	1	40	2
**5**	*Lespedeza dahurica*	2	50	20
	Ziziphus jujube var.spinosus	1	90	10

Site: Guanzhuang forest farm **Forest type:** *Pinus tabulaeformis* plantation

Protected age: 30 a **Subplot area:** 1 m ×1 m **Mean coverage**: 29%

Investigator: Lin Hou **Date:** 2004.08.24

Subplot No.	Herb species	Individuals	Mean height (cm)	Coverage (%)
**1**	*Bothriochloa ischaemum*	100(branches)	50	10
	Artemisia giraldii	5	40	15
**2**	*Urena lobata*	5	70	20
	Kengia serotina	2	60	10
**3**	*Artemisia gmelinii*	5	60	15
**4**	*Patrinia heterophylla*	2	50	3
	Saussurea morifolia	8	45	30
**5**	*Saussurea nivea*	6	80	25

Site: Guanzhuang forest farm **Forest type:** *Pinus tabulaeformis* plantation

Protected age: 30 a **Plot area:** 20 m ×20 m **Canopy density**: 0.50

Altitude: 1163 m **Direction:** South **Gradient:** 25° **Position:** Upper

Plot No. : 11 **Investigator:** Lin Hou **Date:** 2004.08.25

No.	Tree species	DBH/ground diameter（cm）	H (m)	Remark
1	*Betula platyphylla*	2.8	3.4	
2	*Pinus tabulaeformis*	16.5	19.5	
3	*Pinus tabulaeformis*	16.8	19.8	
4	*Prunus davidiana*	2.4	3.0	
5	*Pinus tabulaeformis*	18.4	20.5	
6	*Pinus tabulaeformis*	8.8	11.4	
7	*Quercus Liaotungensis*	3.2	4.4	
8	*Pinus tabulaeformis*	18.5	20.8	
9	*Pinus tabulaeformis*	19.0	21.5	
10	*Pinus tabulaeformis*	5.0	1.8	

11	*Pinus tabulaeformis*	2.8	0.3	
12	*Pinus tabulaeformis*	7.6	10.2	
13	*Pinus tabulaeformis*	16.5	18.2	
14	*Pinus tabulaeformis*	13.8	16.2	
15	*Conus walteri wanger*	3.4	5.2	
16	*Pinus tabulaeformis*	9.4	12.8	
17	*Pinus tabulaeformis*	16.9	19.0	
18	*Pinus tabulaeformis*	6.5	9.8	
19	*Pinus tabulaeformis*	15.5	17.8	
20	*Pinus tabulaeformis*	10.4	13.8	
21	*Pinus tabulaeformis*	5.6	2.0	
22	*Syringa oblata*	5.2	1.9	
23	*Pinus tabulaeformis*	19.2	20.8	
24	*Pinus tabulaeformis*	9.0	12.5	
25	*Pinus tabulaeformis*	9.2	12.5	
26	*Pinus tabulaeformis*	48.0	31.4	
27	*Betula platyphylla*	4.0	5.8	
28	*Pinus tabulaeformis*	5.8	1.9	
29	*Pinus tabulaeformis*	7.4	9.6	
30	*Pinus tabulaeformis*	14.5	18.4	
31	*Pinus tabulaeformis*	7.6	9.2	
32	*Pinus tabulaeformis*	7.5	9.4	
33	*Prunus tomenosa*	2.8	3.4	
34	*Pinus tabulaeformis*	13.8	16.5	
35	*Pinus tabulaeformis*	10.8	15.2	
36	*Pinus tabulaeformis*	5.4	1.8	
37	*Pinus tabulaeformis*	3.8	0.3	
38	*Pinus tabulaeformis*	6.5	9.6	
39	*Quercus Liaotungensis*	4.6	6.4	
40	*Pinus tabulaeformis*	14.5	16.5	
41	*Pinus tabulaeformis*	6.8	8.2	
42	*Pinus tabulaeformis*	13.0	16.5	
43	*Pinus tabulaeformis*	13.2	16.5	
44	*Pinus tabulaeformis*	6.2	8.4	
45	*Pinus tabulaeformis*	14.5	15.2	
46	*Pinus tabulaeformis*	14.0	14.8	
47	*Pinus tabulaeformis*	6.5	8.5	
48	*Pinus tabulaeformis*	12.4	13.8	
49	*Quercus Liaotungensis*	4.4	6.2	

50	*Pinus tabulaeformis*	12.6	13.5	
51	*Pinus tabulaeformis*	12.8	13.5	

Site: Guanzhuang forest farm **Forest type:** *Pinus tabulaeformis* plantation

Protected age: 30 a **Subplot area:** 2 m ×2 m **Mean coverage**: 32%

Investigator: Lin Hou **Date:** 2004.08.26

Subplot No.	Shrub species	Individuals	Mean height (cm)	Coverage (%)
1	*Lespedeza dahurica*	3	45	30
	Rosa hugonis	1	120	3
2	*Lespedeza dahurica*	2	50	25
	Spiraea fritschiana	2	80	10
3	*Lespedeza dahurica*	4	35	20
	Rosa hugonis	2	80	2
4	*Lespedeza dahurica*	3	40	25
	Ziziphus jujube var.*spinosus*	1	110	10
	Rosa hugonis	1	110	2
5	*Lespedeza dahurica*	2	30	15
	Spiraea fritschiana	1	110	15
	Sophora viciifolia	2	80	2

Site: Guanzhuang forest farm **Forest type:** *Pinus tabulaeformis* plantation

Protected age: 30 a **Subplot area:** 1 m ×1 m **Mean coverage**: 27%

Investigator: Lin Hou **Date:** 2004.08.26

Subplot No.	Herb species	Individuals	Mean height (cm)	Coverage (%)
1	*Bothriochloa ischaemum*	800(branches)	40	30
2	*Viola selkirkii*	10	12	15
	Saussurea petrovii	6	15	20
3	*Vicia unijuga*	60	5	15
	Kengia serotina	3	60	15
4	*Viola chaerophylloides*	5	15	15
5	*Bothriochloa ischaemum*	300(branches)	35	20
	Patrinia heterophylla	2	60	4

Site: Wazijie forest farm **Forest type:** *Pinus tabulaeformis* plantation

Protected age: 45 a **Plot area:** 20 m ×20 m **Canopy density**: 0.60

Altitude: 1170 m **Direction:** North **Gradient:** 24° **Position:** Upper

Plot No. : 12 **Investigator:** Lin Hou **Date:** 2004.08.28

NO.	Tree species	DBH/ground diameter（cm）	H (m)	Remark
1	*Pinus tabulaeformis*	20.5	19.4	
2	*Pinus tabulaeformis*	11.8	10.8	
3	*Pinus tabulaeformis*	12.8	12.0	
4	*Betula platyphylla*	2.8	4.2	
5	*Pinus tabulaeformis*	2.8	0.3	
6	*Pinus tabulaeformis*	20.8	19.2	
7	*Pinus tabulaeformis*	11.9	10.9	
8	*Pinus tabulaeformis*	15.4	12.8	
9	*Quercus Liaotungensis*	3.6	4.8	
10	*Pinus tabulaeformis*	18.2	14.0	
11	*Pinus tabulaeformis*	5.8	1.9	
12	*Pinus tabulaeformis*	12.8	11.5	
13	*Pinus tabulaeformis*	20.4	18.0	
14	*Pinus tabulaeformis*	11.6	10.8	
15	*Betula platyphylla*	3.0	4.5	
16	*Pinus tabulaeformis*	23.4	20.2	
17	*Pinus tabulaeformis*	16.3	13.9	
18	*Pinus tabulaeformis*	22.6	19.0	
19	*Pinus tabulaeformis*	22.8	19.0	
20	*Pinus tabulaeformis*	11.2	11.8	
21	*Syringa oblata*	4.0	2.8	
22	*Pinus tabulaeformis*	20.4	15.2	
23	*Pinus tabulaeformis*	58.4	24.8	
24	*Pinus tabulaeformis*	2.5	0.3	
25	*Pinus tabulaeformis*	22.8	20.0	
26	*Pinus tabulaeformis*	13.8	12.0	
27	*Quercus Liaotungensis*	3.5	4.6	
28	*Pinus tabulaeformis*	21.2	20.8	
29	*Pinus tabulaeformis*	11.8	12.2	
30	*Pinus tabulaeformis*	13.8	12.5	
31	*Pinus tabulaeformis*	19.8	15.2	

32	*Pinus tabulaeformis*	19.6	15.2	
33	*Pinus tabulaeformis*	14.2	12.9	
34	*Pinus tabulaeformis*	20.2	15.5	
35	*Pinus tabulaeformis*	14.2	12.8	
36	*Quercus Liaotungensis*	4.0	5.2	

Site: Wazijie forest farm **Forest type:** *Pinus tabulaeformis* plantation

Protected age: 45 a **Subplot area:** 2 m ×2 m **Mean coverage**: 37%

Investigator: Lin Hou **Date:** 2004.08.29

Subplot No.	Shrub species	Individuals	Mean height (cm)	Coverage (%)
**1**	*Lespedeza dahurica*	4	50	15
	Acer ginnala	2	300	10
	Lonicera maccki	1	220	10
	Rubus corchorifolius	1	180	5
**2**	*Lespedeza dahurica*	5	60	20
	Lonicera maccki	1	240	10
	Rubus corchorifolius	2	90	4
	Acer ginnala	3	420	15
	Spiraea fritschiana	1	180	15
**3**	*Lespedeza dahurica*	3	50	10
	Acer ginnala	3	400	15
	Rubus corchorifolius	5	80	5
	Lespedeza dahurica	5	45	10
	Lonicera maccki	2	140	6
**4**	*Lespedeza dahurica*	3	40	10
	Acer ginnala	2	360	10
	Rubus corchorifolius	3	140	5
	Spiraea fritschiana	1	140	9
**5**	*Lespedeza dahurica*	2	50	8
	Lonicera maccki	3	150	7
	Acer ginnala	1	280	8

Site: Wazijie forest farm **Forest type:** *Pinus tabulaeformis* plantation

Protected age: 45 a **Subplot area:** 1 m ×1 m **Mean coverage**: 40%

Investigator: Lin Hou **Date:** 2004.08.29

Subplot No.	Herb species	Individuals	Mean height (cm)	Coverage (%)
**1**	*Bothriochloa ischaemum*	400(branches)	40	25
	Kengia serotina	10	40	20
**2**	*Bothriochloa ischaemum*	350(branches)	60	23
	Kengia serotina	15	50	23
**3**	*Bothriochloa ischaemum*	500(branches)	50	30
	Agrimonia pilosa	5	20	15
**4**	*Bothriochloa ischaemum*	300(branches)	40	20
	Kengia serotina	2	45	10
**5**	*Bothriochloa ischaemum*	400(branches)	50	25
	Urena lobata	1	80	5

Site: Wazijie forest farm **Forest type:** *Pinus tabulaeformis* plantation

Protected age: 45 a **Plot area:** 20 m ×20 m **Canopy density**: 0.70

Altitude: 1160 m **Direction:** North **Gradient:** 22.3° **Position:** Middle

Plot No. : 13 **Investigator:** Lin Hou **Date:** 2004.08.30

No.	Tree species	DBH/ground diameter（cm）	H (m)	Remark
1	*Pinus tabulaeformis*	2.8	0.3	
2	*Pinus tabulaeformis*	18.4	17.2	
3	*Pinus tabulaeformis*	11.8	9.4	
4	*Betula platyphylla*	2.8	3.2	
5	*Pinus tabulaeformis*	5.8	1.8	
6	*Pinus tabulaeformis*	19.8	17.5	
7	*Pinus tabulaeformis*	20.4	17.2	
8	*Pinus tabulaeformis*	2.4	0.2	
9	*Quercus Liaotungensis*	4.0	5.5	
10	*Pinus tabulaeformis*	20.8	17.2	
11	*Pinus tabulaeformis*	17.8	16.4	
12	*Pinus tabulaeformis*	16.5	15.0	

13	*Pinus tabulaeformis*	5.9	1.9	
14	*Pinus tabulaeformis*	52.0	22.4	
15	*Betula platyphylla*	2.0	2.8	
16	*Pinus tabulaeformis*	14.8	13.6	
17	*Pinus tabulaeformis*	11.9	9.6	
18	*Pinus tabulaeformis*	11.4	9.5	
19	*Pinus tabulaeformis*	17.8	15.6	
20	*Pinus tabulaeformis*	18.6	17.0	
21	*Quercus Liaotungensis*	3.0	4.0	
22	*Pinus tabulaeformis*	15.5	14.0	
23	*Pinus tabulaeformis*	20.2	18.0	
24	*Pinus tabulaeformis*	20.6	17.0	
25	*Pinus tabulaeformis*	15.0	13.5	
26	*Pinus tabulaeformis*	2.6	0.2	
27	*Syringa oblata*	3.5	4.8	
28	*Pinus tabulaeformis*	21.4	18.2	
29	*Pinus tabulaeformis*	11.2	9.8	
30	*Pinus tabulaeformis*	20.8	18.4	
31	*Quercus Liaotungensis*	3.8	5.4	
32	*Pinus tabulaeformis*	20.6	18.0	
33	*Pinus tabulaeformis*	11.8	9.5	
34	*Pinus tabulaeformis*	20.8	17.2	
35	*Pinus tabulaeformis*	21.8	17.0	
36	*Betula platyphylla*	4.5	6.5	
37	*Pinus tabulaeformis*	11.9	9.5	
38	*Pinus tabulaeformis*	20.4	17.5	
39	*Pinus tabulaeformis*	20.6	17.5	

Site: Wazijie forest farm **Forest type:** *Pinus tabulaeformis* plantation

Protected age: 45 a **Subplot area:** 2 m ×2 m **Mean coverage**: 27%

Investigator: Lin Hou **Date:** 2004.08.31

Subplot No.	Shrub species	Individuals	Mean height (cm)	Coverage (%)
1	*Lespedeza dahurica*	4	50	15
	Acer ginnala	2	300	10
	Lonicera maccki	1	220	10
	Rubus corchorifolius	1	180	5
2	*Lespedeza dahurica*	5	60	20
	Lonicera maccki	1	240	10
	Rubus corchorifolius	2	90	4
	Acer ginnala	3	420	15
	Spiraea fritschiana	1	180	15
3	*Lespedeza dahurica*	3	50	10
	Acer ginnala	3	400	15
	Rubus corchorifolius	5	80	5
	Lespedeza dahurica	5	45	10
	Lonicera maccki	2	140	6
4	*Lespedeza dahurica*	3	40	10
	Acer ginnala	2	360	10
	Rubus corchorifolius	3	140	5
	Spiraea fritschiana	1	140	9
5	*Lespedeza dahurica*	2	50	8
	Lonicera maccki	3	150	7
	Acer ginnala	1	280	8

Site: Wazijie forest farm **Forest type:** *Pinus tabulaeformis* plantation

Protected age: 45 a **Subplot area:** 1 m ×1 m **Mean coverage**: 29%

Investigator: Lin Hou **Date:** 2004.08.31

Subplot No.	Herb species	Individuals	Mean height (cm)	Coverage (%)
1	*Bothriochloa ischaemum*	300(branches)	50	20
2	*Bothriochloa ischaemum*	500(branches)	50	30
	Kengia serotina	6	60	15
3	*Bothriochloa ischaemum*	350(branches)	45	20
	Kengia serotina	4	60	10
	Spodiopogon sibiricus	3	100	5
4	*Bothriochloa ischaemum*	380(branches)	22	30
5	*Bothriochloa ischaemum*	500(branches)	20	30

Site: Wazijie forest farm **Forest type:** *Pinus tabulaeformis* plantation

Protected age: 45 a **Plot area:** 20 m ×20 m **Canopy density**: 0.60

Altitude: 1175 m **Direction:** North **Gradient:** 21° **Position:** Upper

Plot No. : 14 **Investigator:** Lin Hou **Date:** 2004.09.01

No.	Tree species	DBH/ground diameter（cm）	H (m)	Remark
1	*Betula platyphylla*	3.2	4.0	
2	*Pinus tabulaeformis*	2.4	0.3	
3	*Pinus tabulaeformis*	11.6	10.5	
4	*Syringa oblata*	5.0	6.5	
5	*Pinus tabulaeformis*	19.5	13.0	
6	*Pinus tabulaeformis*	18.5	12.0	
7	*Pinus tabulaeformis*	5.6	1.9	
8	*Pinus tabulaeformis*	19.0	12.0	
9	*Pinus tabulaeformis*	19.5	12.5	
10	*Pinus tabulaeformis*	2.8	0.3	
11	*Pinus tabulaeformis*	22.0	14.0	
12	*Betula platyphylla*	4.8	7.5	
13	*Pinus tabulaeformis*	11.8	10.0	
14	*Pinus tabulaeformis*	19.0	13.0	
15	*Pinus tabulaeformis*	18.5	12.5	
16	*Pinus tabulaeformis*	82.0	24.5	
17	*Pinus tabulaeformis*	22.5	14.5	
18	*Pinus tabulaeformis*	23.0	15.0	
19	*Quercus Liaotungensis*	6.0	8.4	
20	*Pinus tabulaeformis*	18.5	13.5	
21	*Pinus tabulaeformis*	11.2	9.4	
22	*Pinus tabulaeformis*	24.0	15.0	
23	*Pinus tabulaeformis*	21.0	14.0	
24	*Pinus tabulaeformis*	20.5	14.0	
25	*Pinus tabulaeformis*	11.8	9.5	
26	*Betula platyphylla*	7.0	8.5	
27	*Pinus tabulaeformis*	25.0	15.0	
28	*Pinus tabulaeformis*	23.0	15.0	
29	*Pinus tabulaeformis*	16.8	12.0	
30	*Pinus tabulaeformis*	10.8	9.8	
31	*Quercus Liaotungensis*	4.5	6.5	

Site: Wazijie forest farm **Forest type:** *Pinus tabulaeformis* plantation

Protected age: 45 a **Subplot area:** 2 m ×2 m **Mean coverage**: 23%

Investigator: Lin Hou **Date:** 2004.09.02

Subplot No.	Shrub species	Individuals	Mean height (cm)	Coverage (%)
1	*Lespedeza dahurica*	1	60	5
	Rubus corchorifolius	4	90	3
	Lonicera maccki	6	90	3
	Acer ginnala	3	180	5
2	*Lespedeza dahurica*	3	40	8
	Rubus corchorifolius	1	220	5
	Lonicera maccki	2	120	5
	Spiraea fritschiana	3	100	5
	Acer ginnala	2	220	6
3	*Lespedeza dahurica*	2	50	7
	Rubus corchorifolius	2	70	2
	Acer ginnala	3	240	8
	Lonicera maccki	1	90	1
4	*Lespedeza dahurica*	3	40	6
	Rubus corchorifolius	1	50	1
	Lonicera maccki	3	140	7
	Acer ginnala	1	180	3
	Spiraea fritschiana	3	120	8
5	*Lespedeza dahurica*	5	36	10
	Rubus corchorifolius	4	90	4
	Acer ginnala	1	80	1
	Lonicera maccki	5	120	8
	Spiraea fritschiana	1	160	3

Site: Wazijie forest farm **Forest type:** *Pinus tabulaeformis* plantation

Protected age: 45 a **Subplot area:** 1 m ×1 m **Mean coverage**: 32%

Investigator: Lin Hou **Date:** 2004.09.02

Subplot No.	Herb species	Individuals	Mean height (cm)	Coverage (%)
**1**	*Bothriochloa ischaemum*	300(branches)	50	25
	Kengia serotina	3	50	5
**2**	*Bothriochloa ischaemum*	350(branches)	60	30
**3**	*Bothriochloa ischaemum*	600(branches)	40	35
	Anaphalis margaritacea	8	40	10
**4**	*Bothriochloa ischaemum*	500(branches)	50	30
	Potentilla discolor	5	20	10
**5**	*Bothriochloa ischaemum*	300(branches)	45	25

Site: Wazijie forest farm **Forest type:** *Pinus tabulaeformis* plantation

Protected age: 45 a **Plot area:** 20 m ×20 m **Canopy density**: 0.70

Altitude: 1163 m **Direction:** North **Gradient:** 23° **Position:** Middle

Plot No. : 15 **Investigator:** Lin Hou **Date:** 2004.09.03

No.	Tree species	DBH/ground diameter（cm）	H (m)	Remark
1	*Pinus tabulaeformis*	18.5	13.5	
2	*Pinus tabulaeformis*	2.6	0.3	
3	*Pinus tabulaeformis*	5.8	1.9	
4	*Pinus tabulaeformis*	17.5	13.0	
5	*Pinus tabulaeformis*	19.0	14.0	
6	*Betula platyphylla*	3.5	4.5	
7	*Pinus tabulaeformis*	2.8	0.3	
8	*Quercus Liaotungensis*	4.0	5.0	
9	*Pinus tabulaeformis*	18.5	13.0	
10	*Pinus tabulaeformis*	11.8	9.5	
11	*Pinus tabulaeformis*	5.6	1.7	
12	*Pinus tabulaeformis*	18.5	11.0	
13	*Pinus tabulaeformis*	19.0	10.8	
14	*Pinus tabulaeformis*	19.5	11.0	
15	*Pinus tabulaeformis*	75.0	23.5	
16	*Pinus tabulaeformis*	20.8	13.5	
17	*Syringa oblata*	5.0	6.0	
18	*Pinus tabulaeformis*	11.8	10.0	
19	*Pinus tabulaeformis*	19.5	15.5	
20	*Pinus tabulaeformis*	19.8	16.0	
21	*Pinus tabulaeformis*	11.5	9.0	
22	*Pinus tabulaeformis*	22.5	14.0	

23	*Pinus tabulaeformis*	22.0	14.0	
24	*Pinus tabulaeformis*	10	9.5	
25	*Pinus tabulaeformis*	20.8	13.0	
26	*Betula platyphylla*	3.5	4.0	
27	*Pinus tabulaeformis*	21.0	13.5	
28	*Pinus tabulaeformis*	23.5	14.0	
29	*Pinus tabulaeformis*	23.0	14.0	
30	*Quercus Liaotungensis*	3.5	4.5	

Site: Wazijie forest farm **Forest type:** *Pinus tabulaeformis* plantation

Protected age: 45 a **Subplot area:** 2 m ×2 m **Mean coverage**: 23%

Investigator: Lin Hou **Date:** 2004.09.04

Subplot No.	Shrub species	Individuals	Mean height (cm)	Coverage (%)
1	*Lespedeza dahurica*	2	60	5
	Rubus corchorifolius	3	130	5
	Lonicera maccki	2	160	7
	Spiraea fritschiana	2	160	8
	Acer ginnala	2	120	3
2	*Lespedeza dahurica*	3	60	8
	Rubus corchorifolius	3	120	5
	Spiraea fritschiana	1	80	2
	Acer ginnala	3	90	3
3	*Lespedeza dahurica*	4	45	10
	Rubus corchorifolius	1	50	1
	Lonicera maccki	1	220	9
	Acer ginnala	4	80	2
	Spiraea fritschiana	1	60	1
4	*Lespedeza dahurica*	3	50	8
	Rubus corchorifolius	4	120	5
	Spiraea fritschiana	3	100	10
	Acer ginnala	2	100	3
5	*Lespedeza dahurica*	2	40	5
	Lonicera maccki	3	140	5
	Acer ginnala	1	60	1
	Spiraea fritschiana	2	80	3
	Rubus corchorifolius	5	110	5

Site: Wazijie forest farm **Forest type:** *Pinus tabulaeformis* plantation

Protected age: 45 a **Subplot area:** 1 m ×1 m **Mean coverage**: 26%

Investigator: Lin Hou **Date:** 2004.09.04

Subplot No.	Herb species	Individuals	Mean height (cm)	Coverage (%)
**1**	*Bothriochloa ischaemum*	600(branches)	30	30
**2**	*Bothriochloa ischaemum*	400(branches)	40	25
**3**	*Bothriochloa ischaemum*	300(branches)	40	25
	Kengia serotina	5	45	10
**4**	*Bothriochloa ischaemum*	250(branches)	35	20
**5**	*Bothriochloa ischaemum*	300(branches)	30	20

Site: Wazijie forest farm **Forest type:** *Pinus tabulaeformis* plantation

Protected age: 45 a **Plot area:** 20 m ×20 m **Canopy density**: 0.70

Altitude: 1163 m **Direction:** North **Gradient:** 26° **Position:** Middle

Plot No. : 16 **Investigator:** Lin Hou **Date:** 2004.09.05

No.	Tree species	DBH/ground diameter（cm）	H (m)	Remark
1	*Syringa oblata*	3.2	4.0	
2	*Pinus tabulaeformis*	2.8	0.3	
3	*Pinus tabulaeformis*	20.4	13.5	
4	*Pinus tabulaeformis*	20.5	13.5	
5	*Pinus tabulaeformis*	10.8	9.5	
6	*Pinus tabulaeformis*	9.5	9.3	
7	*Pinus tabulaeformis*	18.5	13.0	
8	*Betula platyphylla*	3.5	4.2	
9	*Pinus tabulaeformis*	18.5	13.0	
10	*Pinus tabulaeformis*	18.5	12.8	
11	*Pinus tabulaeformis*	2.6	0.3	
12	*Pinus tabulaeformis*	9.0	9.5	
13	*Pinus tabulaeformis*	12.5	11.0	
14	*Quercus Liaotungensis*	5.0	6.5	
15	*Pinus tabulaeformis*	5.8	1.8	
16	*Pinus tabulaeformis*	13.0	11.5	
17	*Pinus tabulaeformis*	20.8	13.5	
18	*Pinus tabulaeformis*	21.5	14.5	
19	*Pinus tabulaeformis*	9.5	9.2	

20	*Pinus tabulaeformis*	13.5	12.5	
21	*Pinus tabulaeformis*	75.0	27.0	
22	*Quercus Liaotungensis*	6.5	8.0	
23	*Pinus tabulaeformis*	13.0	11.8	
24	*Pinus tabulaeformis*	21.0	13.0	
25	*Pinus tabulaeformis*	21.0	13.5	
26	*Pinus tabulaeformis*	11.8	9.5	
27	*Pinus tabulaeformis*	20.5	12.0	
28	*Pinus tabulaeformis*	20.5	13.5	

Site: Wazijie forest farm **Forest type:** *Pinus tabulaeformis* plantation

Protected age: 45 a **Subplot area:** 2 m ×2 m **Mean coverage**: 26%

Investigator: Lin Hou **Date:** 2004.09.06

Subplot No.	Shrub species	Individuals	Mean height (cm)	Coverage (%)
**1**	*Lespedeza dahurica*	1	30	2
	Lonicera maccki	2	150	5
	Acer ginnala	3	80	3
**2**	*Lespedeza dahurica*	3	30	4
	Spiraea fritschiana	1	120	3
	Acer ginnala	2	120	5
	Lonicera maccki	1	60	1
	Rubus corchorifolius	2	90	2
**3**	*Lespedeza dahurica*	2	60	8
	Rubus corchorifolius	3	140	6
	Lonicera maccki	1	50	1
**4**	*Lespedeza dahurica*	3	50	7
	Rubus corchorifolius	1	80	1
	Acer ginnala	1	180	3
	Spiraea fritschiana	2	90	5
**5**	*Lespedeza dahurica*	2	45	7
	Rubus corchorifolius	5	90	4
	Acer ginnala	1	60	1
	Spiraea fritschiana	1	180	13

Site: Wazijie forest farm **Forest type:** *Pinus tabulaeformis* plantation

Protected age: 45 a **Subplot area:** 1 m ×1 m **Mean coverage**: 28%

Investigator: Lin Hou **Date:** 2004.09.06

Subplot No.	Herb species	Individuals	Mean height (cm)	Coverage (%)
**1**	*Bothriochloa ischaemum*	600(branches)	40	30
	Kengia serotina	3	50	5
**2**	*Bothriochloa ischaemum*	400(branches)	50	25
	Viola japonica var. stenopetala	3	10	5
**3**	*Bothriochloa ischaemum*	600(branches)	35	30
**4**	*Bothriochloa ischaemum*	300(branches)	25	22
**5**	*Bothriochloa ischaemum*	500(branches)	30	30

Site: Wazijie forest farm **Forest type:** *Pinus tabulaeformis* plantation

Protected age: 45 a **Plot area:** 20 m ×20 m **Canopy density**: 0.70

Altitude: 1120 m **Direction:** **Gradient:** 0° **Position:** Gully bottom

Plot No. : 17 **Investigator:** Lin Hou **Date:** 2004.09.07

No.	Tree species	DBH/ground diameter（cm）	H (m)	Remark
1	*Pinus tabulaeformis*	2.8	1.6	
2	*Betula platyphylla*	3.8	4.2	
3	*Pinus tabulaeformis*	10.0	9.0	
4	*Pinus tabulaeformis*	14.5	12.8	
5	*Pinus tabulaeformis*	11.5	9.5	
6	*Pinus tabulaeformis*	14.5	12.5	
7	*Betula platyphylla*	4.0	5.5	
8	*Pinus tabulaeformis*	21.0	13.0	
9	*Pinus tabulaeformis*	22.0	13.0	
10	*Quercus Liaotungensis*	4.5	7.5	
11	*Pinus tabulaeformis*	2.5	1.7	
12	*Betula platyphylla*	4.0	6.0	
13	*Pinus tabulaeformis*	12.5	11.5	
14	*Quercus Liaotungensis*	5.5	8.0	
15	*Pinus tabulaeformis*	5.8	1.9	
16	*Pinus tabulaeformis*	22.5	13.5	
17	*Betula platyphylla*	3.5	4.0	
18	*Pinus tabulaeformis*	13.0	11.5	

19	*Pinus tabulaeformis*	70.0	24.5	
20	*Syringa oblata*	3.8	4.5	
21	*Pinus tabulaeformis*	20.5	12.8	
22	*Pinus tabulaeformis*	11.8	9.5	
23	*Pinus tabulaeformis*	21.0	13.0	
24	*Quercus Liaotungensis*	4.0	5.5	
25	*Pinus tabulaeformis*	16.0	12.0	
26	*Pinus tabulaeformis*	20.5	11.8	
27	*Pinus tabulaeformis*	11.5	11.0	
28	*Pinus tabulaeformis*	15.0	12.5	
29	*Pinus tabulaeformis*	20.5	12.8	
30	*Pinus tabulaeformis*	21.5	13.0	
31	*Pinus tabulaeformis*	10.5	9.5	
32	*Syringa oblata*	2.6	3.2	
33	*Pinus tabulaeformis*	22.0	13.5	
34	*Pinus tabulaeformis*	25.5	14.5	
35	*Pinus tabulaeformis*	21.5	13.0	
36	*Pinus tabulaeformis*	14.0	12.5	
37	*Pinus tabulaeformis*	15.8	12.4	
38	*Pinus tabulaeformis*	11.0	9.5	

Site: Wazijie forest farm **Forest type:** *Pinus tabulaeformis* plantation

Protected age: 45 a **Subplot area:** 2 m ×2 m **Mean coverage**: 14%

Investigator: Lin Hou **Date:** 2004.09.08

**Subplot No.**	**Shrub species**	**Individuals**	**Mean height (cm)**	**Coverage (%)**
**1**	*Lespedeza dahurica*	2	60	5
	Spiraea fritschiana	2	80	2
	Rubus corchorifolius	3	80	4
	Lonicera maccki	2	90	2
**2**	*Lespedeza dahurica*	4	45	10
	Spiraea fritschiana	1	60	1
	Acer ginnala	4	70	3
	Rubus corchorifolius	3	150	6
**3**	*Lespedeza dahurica*	2	40	5
	Spiraea fritschiana	1	90	1
	Lonicera maccki	2	80	2

**4**	*Lespedeza dahurica*	6	30	10
	Rubus corchorifolius	1	45	1
	Acer ginnala	2	90	3
	Lonicera maccki	5	140	5
**5**	*Lespedeza dahurica*	1	30	2
	Rubus corchorifolius	5	80	7
	Spiraea fritschiana	2	140	6
	Acer ginnala	1	240	5

Site: Wazijie forest farm **Forest type:** *Pinus tabulaeformis* plantation

Protected age: 45 a **Subplot area:** 1 m ×1 m **Mean coverage**: 27%

Investigator: Lin Hou **Date:** 2004.09.08

Subplot No.	Herb species	Individuals	Mean height (cm)	Coverage (%)
**1**	*Bothriochloa ischaemum*	400(branches)	45	30
**2**	*Bothriochloa ischaemum*	350(branches)	25	20
	Neottianthe cucullata	3	20	10
**3**	*Bothriochloa ischaemum*	300(branches)	50	25
**4**	*Bothriochloa ischaemum*	250(branches)	30	20
**5**	*Bothriochloa ischaemum*	300(branches)	35	30

Site: Wazijie forest farm **Forest type:** *Pinus tabulaeformis* plantation

Protected age: 45 a **Plot area:** 20 m ×20 m **Canopy density**: 0.60

Altitude: 1130 m **Direction:** North **Gradient:** 10° **Position:** Lower

Plot No. : 18 **Investigator:** Lin Hou **Date:** 2004.09.09

No.	Tree species	DBH/ground diameter（cm）	H (m)	Remark
1	*Pinus tabulaeformis*	16.0	13.5	
2	*Pinus tabulaeformis*	16.5	13.5	
3	*Pinus tabulaeformis*	11.5	9.8	
4	*Betula platyphylla*	4.5	6.0	
5	*Pinus tabulaeformis*	5.6	1.8	
6	*Pinus tabulaeformis*			
7	*Pinus tabulaeformis*	2.6	0.3	
8	*Pinus tabulaeformis*	14.5	13.8	

9	*Pinus tabulaeformis*	15.0	14.5	
10	*Pinus tabulaeformis*	11.4	10.5	
11	*Quercus Liaotungensis*	4.0	5.5	
12	*Pinus tabulaeformis*	12.5	11.4	
13	*Pinus tabulaeformis*	11.8	10.7	
14	*Pinus tabulaeformis*	5.9	1.9	
15	*Pinus tabulaeformis*	13.6	11.6	
16	*Betula platyphylla*	6.0	8.5	
17	*Pinus tabulaeformis*	11.4	10.3	
18	*Pinus tabulaeformis*	21.0	16.0	
19	*Pinus tabulaeformis*	14.5	12.0	
20	*Syringa oblata*	3.5	5.2	
21	*Pinus tabulaeformis*	70.0	32.0	
22	*Pinus tabulaeformis*	15.2	14.5	
23	*Pinus tabulaeformis*	20.5	14.8	
24	*Quercus Liaotungensis*	4.0	6.0	
25	*Pinus tabulaeformis*	11.6	10.5	
26	*Pinus tabulaeformis*	2.8	0.3	
27	*Pinus tabulaeformis*	20.4	15.8	
28	*Pinus tabulaeformis*	21.0	15.6	
29	*Pinus tabulaeformis*	20.5	16.0	
30	*Pinus tabulaeformis*	21.0	16.0	
31	*Pinus tabulaeformis*	17.5	13.8	
32	*Pinus tabulaeformis*	20.4	15.8	
33	*Pinus tabulaeformis*	20.5	15.5	
34	*Pinus tabulaeformis*	21.0	16.0	
35	*Pinus tabulaeformis*	21.5	16.0	

Site: Wazijie forest farm **Forest type:** *Pinus tabulaeformis* plantation

Protected age: 45 a **Subplot area:** 2 m ×2 m **Mean coverage**: 16%

Investigator: Lin Hou **Date:** 2004.09.10

Subplot No.	Shrub species	Individuals	Mean height (cm)	Coverage (%)
1	*Lespedeza dahurica*	4	50	10
	Rubus corchorifolius	1	70	1
	Lonicera maccki	6	110	7
	Acer ginnala	2	60	2

	Spiraea fritschiana	3	80	6
2	*Lespedeza dahurica*	1	60	4
	Rubus corchorifolius	2	110	4
	Spiraea fritschiana	2	120	5
	Acer ginnala	1	90	2
3	*Lespedeza dahurica*	2	45	6
	Rubus corchorifolius	4	60	3
	Acer ginnala	3	120	5
	Lonicera maccki	3	160	8
4	*Lespedeza dahurica*	3	45	8
	Rubus corchorifolius	2	70	2
	Spiraea fritschiana	1	150	3
5	*Rubus corchorifolius*	1	55	1
	Spiraea fritschiana	3	100	3

Site: Wazijie forest farm **Forest type:** *Pinus tabulaeformis* plantation

Protected age: 45 a **Subplot area:** 1 m ×1 m **Mean coverage**: 24%

Investigator: Lin Hou **Date:** 2004.09.10

**Subplot No.**	**Herb species**	**Individuals**	**Mean height (cm)**	**Coverage (%)**
1	*Bothriochloa ischaemum*	300(branches)	45	25
	Aster tataricus	3	40	8
2	*Bothriochloa ischaemum*	250(branches)	40	20
3	*Bothriochloa ischaemum*	350(branches)	40	25
4	*Bothriochloa ischaemum*	350(branches)	40	25
5	*Bothriochloa ischaemum*	200(branches)	20	15

Site: Wazijie forest farm **Forest type:** *Pinus tabulaeformis* plantation

Protected age: 45 a **Plot area:** 20 m ×20 m **Canopy density**: 0.70

Altitude: 1150 m **Direction:** North **Gradient:** 8° **Position:** Ridge top

Plot No. : 19 **Investigator:** Lin Hou **Date:** 2004.09.15

No.	Tree species	DBH/ground diameter（cm）	H (m)	Remark
1	*Quercus Liaotungensis*	4.2	5.5	
2	*Pinus tabulaeformis*	5.8	1.9	
3	*Pinus tabulaeformis*	16.5	14.8	
4	*Pinus tabulaeformis*	16	14.5	
5	*Betula platyphylla*	3.6	4.5	
6	*Pinus tabulaeformis*	21.0	16.6	
7	*Pinus tabulaeformis*	16.0	14.8	
8	*Pinus tabulaeformis*	11.5	10.2	
9	*Pinus tabulaeformis*	16.5	14.4	
10	*Pinus tabulaeformis*	16.0	14.8	
11	*Pinus tabulaeformis*	11.0	10.0	
12	*Syringa oblata*	3.8	4.2	
13	*Pinus tabulaeformis*	75.0	38.0	
14	*Pinus tabulaeformis*	14.5	14.6	
15	*Pinus tabulaeformis*	2.5	0.3	
16	*Quercus Liaotungensis*	4.0	5.4	
17	*Pinus tabulaeformis*	5.6	1.8	
18	*Pinus tabulaeformis*	11.8	10.4	
19	*Pinus tabulaeformis*	14.0	15.2	
20	*Pinus tabulaeformis*	11.4	10.2	
21	*Pinus tabulaeformis*	20.4	15.5	
22	*Pinus tabulaeformis*	16.5	14.8	
23	*Pinus tabulaeformis*	17.0	15.5	
24	*Betula platyphylla*	5.0	6.4	
25	*Pinus tabulaeformis*	2.8	0.3	
26	*Pinus tabulaeformis*	20.6	15.8	
27	*Pinus tabulaeformis*	20.4	15.0	
28	*Pinus tabulaeformis*	20.5	15.0	
29	*Quercus Liaotungensis*	4.8	6.2	
30	*Pinus tabulaeformis*	11.8	10.6	
31	*Pinus tabulaeformis*	20.5	15.0	
32	*Betula platyphylla*			

Site: Wazijie forest farm **Forest type:** *Pinus tabulaeformis* plantation

Protected age: 45 a **Subplot area:** 2 m ×2 m **Mean coverage**: 11%

Investigator: Lin Hou **Date:** 2004.09.16

Subplot No.	Shrub species	Individuals	Mean height (cm)	Coverage (%)
1	*Lespedeza dahurica*	5	40	10
	Spiraea fritschiana	1	180	4
	Acer ginnala	2	160	5
2	*Rubus corchorifolius*	3	70	4
	Acer ginnala	1	100	3
	Spiraea fritschiana	2	120	4
3	*Rubus corchorifolius*	5	90	5
	Spiraea fritschiana	3	100	3
	Acer ginnala	1	120	3
4	*Rubus corchorifolius*	3	120	6
	Spiraea fritschiana	1	80	1
5	*Lespedeza dahurica*	1	40	2
	Rubus corchorifolius	1	60	1
	Spiraea fritschiana	2	90	5

Site: Wazijie forest farm **Forest type:** *Pinus tabulaeformis* plantation

Protected age: 45 a **Subplot area:** 1 m ×1 m **Mean coverage**: 33%

Investigator: Lin Hou **Date:** 2004.09.16

Subplot No.	Herb species	Individuals	Mean height (cm)	Coverage (%)
1	*Bothriochloa ischaemum*	350(branches)	30	35
	Viola yedoensis	3	12	5
2	*Bothriochloa ischaemum*	300(branches)	35	30
3	*Bothriochloa ischaemum*	250(branches)	30	25
	Kengia serotina	2	50	5
4	*Bothriochloa ischaemum*	300(branches)	40	30
5	*Bothriochloa ischaemum*	250(branches)	50	25
	Viola chaerophylloides	3	15	5

Site: Caijiachuan forest farm **Forest type:** *Pinus tabulaeformis* plantation

Protected age: 60 a **Plot area:** 20 m ×20 m **Canopy density**: 0.60

Altitude: 1200 m **Direction:** Northeast **Gradient:** 19° **Position:** Middle

Plot No. : 20 **Investigator:** Lin Hou **Date:** 2004.09.17

No.	Tree species	DBH/ground diameter（cm）	H (m)	Remark
1	*Pinus tabulaeformis*	1.3	1.6	
2	*Pinus tabulaeformis*	0.5	0.3	
3	*Populus davidiana*	3.2	4.5	
4	*Pinus tabulaeformis*	0.5	0.3	
5	*Pinus tabulaeformis*	0.5	0.3	
6	*Pinus tabulaeformis*	1.5	3.0	
7	*Pinus tabulaeformis*	2.0	1.9	
8	*Pinus tabulaeformis*	12.5	18.5	
9	*Pinus tabulaeformis*	0.5	0.3	
10	*Populus davidiana*	3.0	4.0	
11	*Pinus tabulaeformis*	0.4	0.3	
12	*Pinus tabulaeformis*	0.4	0.3	
13	*Pinus tabulaeformis*	0.4	0.3	
14	*Pinus tabulaeformis*	0.4	0.3	
15	*Pinus tabulaeformis*	12.4	18.0	
16	*Prunus tomenosa*	2.8	3.2	
17	*Pinus tabulaeformis*	12.5	18.5	
18	*Pinus tabulaeformis*	2.6	1.8	
19	*Pinus tabulaeformis*	8.3	11.6	
20	*Pinus tabulaeformis*	8.5	11	
21	*Pinus tabulaeformis*	8.0	11.5	
22	*Pinus tabulaeformis*	8.3	11.6	
23	*Pinus tabulaeformis*	50.0	28.0	
24	*Pinus tabulaeformis*	2.0	1.7	
25	*Pinus tabulaeformis*	8.5	11	
26	*Quercus Liaotungensis*	4.0	6.0	
27	*Pinus tabulaeformis*	12.5	19.0	
28	*Pinus tabulaeformis*	0.5	0.3	
29	*Pinus tabulaeformis*	0.4	0.2	
30	*Pinus tabulaeformis*	9.0	11	
31	*Pinus tabulaeformis*	1.3	0.3	
32	*Pinus tabulaeformis*	0.5	0.2	

33	*Quercus Liaotungensis*	4.5	6.0	
34	*Pinus tabulaeformis*	9.3	11.7	
35	*Pinus tabulaeformis*	9.3	11.6	
36	*Pinus tabulaeformis*	2.0	1.6	
37	*Pinus tabulaeformis*	30.0	36.0	
38	*Pinus tabulaeformis*	9.0	11.8	
39	*Pinus tabulaeformis*	9.0	11	
40	*Pinus tabulaeformis*	1.5	0.3	
41	*Quercus Liaotungensis*	5.5	7.0	
42	*Pinus tabulaeformis*	0.7	0.2	
43	*Pinus tabulaeformis*	0.4	0.2	
44	*Pinus tabulaeformis*	0.5	0.2	
45	*Pinus tabulaeformis*	0.3	0.3	
46	*Pinus tabulaeformis*	0.3	0.3	
47	*Pinus tabulaeformis*	12.5	18.8	
48	*Syringa oblata*	2.8	3.2	
49	*Pinus tabulaeformis*	2.0	1.6	
50	*Pinus tabulaeformis*	0.5	0.2	
51	*Pinus tabulaeformis*	0.4	0.3	
52	*Pinus tabulaeformis*	0.5	0.3	
53	*Pinus tabulaeformis*	8.9	11.0	
54	*Pinus tabulaeformis*	8.7	11.8	
55	*Pinus tabulaeformis*	42.0	30.0	
56	*Pinus tabulaeformis*	2.0	1.5	
57	*Pinus tabulaeformis*	0.4	0.3	
58	*Pinus tabulaeformis*	0.5	0.2	
59	*Pinus tabulaeformis*	0.5	0.2	
60	*Pinus tabulaeformis*	0.5	0.2	
61	*Pinus tabulaeformis*	0.5	0.2	
62	*Toxicodendron vernicifluum*	6.0	4.2	
63	*Pinus tabulaeformis*	0.5	0.3	
64	*Pinus tabulaeformis*	12.5	19.0	
65	*Pinus tabulaeformis*	1.4	2.2	
66	*Pinus tabulaeformis*	0.4	0.2	
67	*Pinus tabulaeformis*	0.6	0.2	
68	*Pinus tabulaeformis*	0.5	0.3	
69	*Pinus tabulaeformis*	2.1	1.9	
70	*Populus davidiana*	3.5	4.8	

Site: Caijiachuan forest farm **Forest type:** *Pinus tabulaeformis* plantation

Protected age: 60 a **Subplot area:** 2 m ×2 m **Mean coverage**: 26%

Investigator: Lin Hou **Date:** 2004.09.18

Subplot No.	Shrub species	Individuals	Mean height (cm)	Coverage (%)
1	*Lespedeza dahurica*	4	60	25
2	*Lespedeza dahurica*	6	50	30
	Ostryopsis davidiana	1	220	10
3	*Lespedeza dahurica*	3	45	20
4	*Lespedeza dahurica*	2	50	15
5	*Acer ginnala*	2	300	20
	Berberis dolichobotrys	1	250	10

Site: Caijiachuan forest farm **Forest type:** *Pinus tabulaeformis* plantation

Protected age: 60 a **Subplot area:** 1 m ×1 m **Mean coverage**: 35%

Investigator: Lin Hou **Date:** 2004.09.18

Subplot No.	Herb species	Individuals	Mean height (cm)	Coverage (%)
1	*Bothriochloa ischaemum*	600(branches)	30	25
	Scutellaria baicalensis	2	40	5
	Kengia serotina	20	45	10
2	*Bothriochloa ischaemum*	400(branches)	45	25
	Bupleurum chinense	1	110	10
	Polygonatum sibircum	1	80	5
	Kengia serotina	5	60	5
3	*Bothriochloa ischaemum*	300(branches)	40	15
	Scutellaria baicalensis	2	45	5
	Artemisia mongolica	3	70	10
4	*Bothriochloa ischaemum*	500(branches)	50	25
	Kengia serotina	15	40	15
5	*Bothriochloa ischaemum*	200(branches)	70	15
	Adenophora stricta	2	100	5

Site: Caijiachuan forest farm **Forest type:** *Pinus tabulaeformis* plantation

Protected age: 60 a **Plot area:** 20 m ×20 m **Canopy density**: 0.40

Altitude: 1155 m **Direction:** North **Gradient:** **5°** **Position:** Ridge top

Plot No. : 21 **Investigator:** Lin Hou **Date:** 2004.09.19

No.	Tree species	DBH/ground diameter（cm）	H (m)	Remark
1	*Pinus tabulaeformis*	2.2	5.5	
2	*Pinus tabulaeformis*	2.0	5.4	
3	*Populus davidiana*	3.0	4.4	
4	*Pinus tabulaeformis*	8.5	18.5	
5	*Pinus tabulaeformis*	10.2	19.5	
6	*Pinus tabulaeformis*	0.3	0.8	
7	*Pinus tabulaeformis*	0.6	0.3	
8	*Pinus tabulaeformis*	0.4	0.2	
9	*Pinus tabulaeformis*	0.5	0.2	
10	*Populus davidiana*	2.8	4.5	
11	*Pinus tabulaeformis*	0.8	0.3	
12	*Pinus tabulaeformis*	0.5	0.2	
13	*Pinus tabulaeformis*	0.6	0.2	
14	*Pinus tabulaeformis*	0.8	0.3	
15	*Pinus tabulaeformis*	0.5	0.2	
16	*Prunus tomenosa*	3.8	4.5	
17	*Pinus tabulaeformis*	10.5	20.0	
18	*Pinus tabulaeformis*	10.0	21.0	
19	*Pinus tabulaeformis*	1.8	5.5	
20	*Pinus tabulaeformis*	1.9	5.5	
21	*Pinus tabulaeformis*	70.0	34.0	
22	*Pinus tabulaeformis*	9.5	17.9	
23	*Pinus tabulaeformis*	0.8	0.3	
24	*Pinus tabulaeformis*	1.1	0.3	
25	*Pinus tabulaeformis*	0.9	0.3	
26	*Pinus tabulaeformis*	0.8	0.3	
27	*Pinus tabulaeformis*	0.6	0.2	
28	*Pinus tabulaeformis*	0.5	0.3	
29	*Pinus tabulaeformis*	0.8	0.2	
30	*Pinus tabulaeformis*	0.5	0.3	
31	*Pinus tabulaeformis*	12.6	21.8	
32	*Pinus tabulaeformis*	12.4	21.5	

33	*Quercus Liaotungensis*	5.2	6.5	
34	*Pinus tabulaeformis*	45.0	38.0	
35	*Pinus tabulaeformis*	10.4	18.5	
36	*Pinus tabulaeformis*	10.8	19.5	
37	*Pinus tabulaeformis*	12.5	21.5	
38	*Pinus tabulaeformis*	0.4	0.2	
39	*Pinus tabulaeformis*	0.5	0.2	
40	*Pinus tabulaeformis*	0.8	0.3	
41	*Quercus Liaotungensis*	3.8	4.5	
42	*Pinus tabulaeformis*	0.6	0.2	
43	*Pinus tabulaeformis*	0.5	0.2	
44	*Pinus tabulaeformis*	13.6	17.8	
45	*Pinus tabulaeformis*	1.8	5.6	
46	*Pinus tabulaeformis*	1.9	5.6	
47	*Pinus tabulaeformis*	50.0	38.0	
48	*Syringa oblata*			
49	*Pinus tabulaeformis*	1.6	5.8	
50	*Pinus tabulaeformis*	1.8	5.9	
51	*Pinus tabulaeformis*	0.5	0.3	
52	*Pinus tabulaeformis*	0.6	0.3	
53	*Pinus tabulaeformis*	0.8	0.3	
54	*Pinus tabulaeformis*	0.5	0.2	
55	*Pinus tabulaeformis*	1.1	0.3	
56	*Pinus tabulaeformis*	13.8	18.4	
57	*Pinus tabulaeformis*	8.4	15.0	
58	*Pinus tabulaeformis*	9.2	11.5	
59	*Pinus tabulaeformis*	13.5	16.5	
60	*Pinus tabulaeformis*	45.0	27.0	
61	*Pinus tabulaeformis*	13.4	15.8	
62	*Toxicodendron vernicifluum*	3.2	4.4	
63	*Pinus tabulaeformis*	10.5	18.4	
64	*Pinus tabulaeformis*	9.5	13.5	
65	*Pinus tabulaeformis*	0.6	0.2	
66	*Pinus tabulaeformis*	0.5	0.3	
67	*Pinus tabulaeformis*	0.6	0.2	
68	*Pinus tabulaeformis*	0.8	0.3	
69	*Pinus tabulaeformis*	13.4	18.5	
70	*Populus davidiana*	2.6	3.4	

Site: Caijiachuan forest farm **Forest type:** *Pinus tabulaeformis* plantation

Protected age: 60 a **Subplot area:** 2 m ×2 m **Mean coverage**: 24%

Investigator: Lin Hou **Date:** 2004.09.20

Subplot No.	Shrub species	Individuals	Mean height (cm)	Coverage (%)
1	*Lespedeza dahurica*	4	60	20
2	*Lespedeza dahurica*	5	50	25
3	*Lespedeza dahurica*	3	50	15
	Acer ginnala	1	280	10
4	*Lespedeza dahurica*	5	35	20
5	*Lespedeza dahurica*	4	60	25

Site: Caijiachuan forest farm **Forest type:** *Pinus tabulaeformis* plantation

Protected age: 60 a **Subplot area:** 1 m ×1 m **Mean coverage**: 34%

Investigator: Lin Hou **Date:** 2004.09.20

Subplot No.	Herb species	Individuals	Mean height (cm)	Coverage (%)
1	*Kengia serotina*	8	50	10
	Spodiopogon sibiricus	1	100	5
	Artemisia mongolica	1	90	5
2	*Bothriochloa ischaemum*	400(branches)	60	20
	Kengia serotina	10	40	10
	Artemisia mongolica	5	50	15
3	*Kengia serotina*	15	45	13
	Bothriochloa ischaemum	600(branches)	40	30
	Potentilla discolor	2	20	5
4	*Kengia serotina*	10	50	10
	Scutellaria baicalensis	1	60	3
	Artemisia mongolica	2	80	10
5	*Bothriochloa ischaemum*	600(branches)	35	25
	Artemisia mongolica	3	75	10

Site: Caijiachuan forest farm **Forest type:** *Pinus tabulaeformis* plantation

Protected age: 60 a **Plot area:** 20 m ×20 m **Canopy density**: 0.40

Altitude: 1150 m **Direction:** North **Gradient:** **18°** **Position:** Upper

Plot No. : 22 **Investigator:** Lin Hou **Date:** 2004.09.21

No.	Tree species	DBH/ground diameter（cm）	H (m)	Remark
1	*Pinus tabulaeformis*	11.8	15.6	
2	*Pinus tabulaeformis*	11.5	15.5	
3	*Pinus tabulaeformis*	11.0	16.0	
4	*Pinus tabulaeformis*	6.5	12.0	
5	*Populus davidiana*	3.2	2.8	
6	*Pinus tabulaeformis*	7.5	9.4	
7	*Pinus tabulaeformis*	19.0	16.8	
8	*Pinus tabulaeformis*	10.8	15.6	
9	*Pinus tabulaeformis*	0.9	0.25	
10	*Pinus tabulaeformis*	0.6	0.3	
11	*Pinus tabulaeformis*	0.5	0.29	
12	*Pinus tabulaeformis*	0.6	0.21	
13	*Pinus tabulaeformis*	0.6	0.3	
14	*Pinus tabulaeformis*	0.8	0.29	
15	*Pinus tabulaeformis*	0.6	0.21	
16	*Pinus tabulaeformis*	0.5	0.22	
17	*Quercus Liaotungensis*	4.2	6.5	
18	*Pinus tabulaeformis*	0.5	0.23	
19	*Pinus tabulaeformis*	3.2	1.5	
20	*Pinus tabulaeformis*	2.8	1.4	
21	*Pinus tabulaeformis*	2.5	1.4	
22	*Pinus tabulaeformis*	86.0	34.0	
23	*Pinus tabulaeformis*	0.5	0.29	
24	*Pinus tabulaeformis*	0.5	0.3	
25	*Pinus tabulaeformis*	0.5	0.3	
26	*Pinus tabulaeformis*	0.5	0.3	
27	*Pinus tabulaeformis*	0.5	0.3	
28	*Pinus tabulaeformis*	0.5	0.3	
29	*Pinus tabulaeformis*	0.5	0.28	
30	*Pinus tabulaeformis*	0.5	0.3	
31	*Pinus tabulaeformis*	0.5	0.3	
32	*Pinus tabulaeformis*	0.5	0.3	
33	*Pinus tabulaeformis*	0.5	0.3	
34	*Pinus tabulaeformis*	0.5	0.3	
35	*Pinus tabulaeformis*	3.0	1.5	
36	*Quercus Liaotungensis*	4.8	5.5	
37	*Pinus tabulaeformis*	3.5	1.6	
38	*Pinus tabulaeformis*	4	1.8	

39	*Pinus tabulaeformis*	4.2	1.8	
40	*Pinus tabulaeformis*	8.5	10.5	
41	*Pinus tabulaeformis*	0.5	0.3	
42	*Pinus tabulaeformis*	0.5	0.3	
43	*Pinus tabulaeformis*	0.5	0.2	
44	*Pinus tabulaeformis*	10.6	15.5	
45	*Pinus tabulaeformis*	10.5	16.0	
46	*Pinus tabulaeformis*	3.4	1.6	
47	*Pinus tabulaeformis*	3.5	1.5	
48	*Pinus tabulaeformis*	3.0	1.5	
49	*Pinus tabulaeformis*	11.4	14.5	
50	*Pinus tabulaeformis*	11.0	14.5	
51	*Pinus tabulaeformis*	11.5	14.5	
52	*Pinus tabulaeformis*	17.6	19.5	
53	*Pinus tabulaeformis*	15.5	17.0	
54	*Pinus tabulaeformis*	60.5	38.0	
55	*Pinus tabulaeformis*	0.5	0.3	
56	*Pinus tabulaeformis*	13.4	16.8	
57	*Pinus tabulaeformis*	13.5	16.5	
58	*Pinus tabulaeformis*	13.6	16.5	
59	*Pinus tabulaeformis*	13.2	17.8	
60	*Pinus tabulaeformis*	10.5	13.4	
61	*Pinus tabulaeformis*	0.5	0.3	
62	*Prunus tomenosa*	3.2	3.6	
63	*Pinus tabulaeformis*	0.5	0.25	
64	*Pinus tabulaeformis*	0.5	0.27	
65	*Pinus tabulaeformis*	0.5	0.3	
66	*Pinus tabulaeformis*	0.5	0.26	
67	*Pinus tabulaeformis*	0.5	0.3	
68	*Pinus tabulaeformis*	65.0	36.0	
69	*Pinus tabulaeformis*	0.5	0.3	
70	*Pinus tabulaeformis*	0.5	0.3	
71	*Pinus tabulaeformis*	0.5	0.3	
72	*Pinus tabulaeformis*	0.5	0.3	
73	*Pinus tabulaeformis*	19.0	18.0	

Site: Caijiachuan forest farm **Forest type:** *Pinus tabulaeformis* plantation

Protected age: 60 a **Subplot area:** 2 m ×2 m **Mean coverage**: 26%

Investigator: Lin Hou **Date:** 2004.09.22

Subplot No.	Shrub species	Individuals	Mean height (cm)	Coverage (%)
1	*Lespedeza dahurica*	3	60	20
	Cotoneaster zbakelii	1	180	10
2	*Lespedeza dahurica*	2	50	15
	Rosa hugonis	2	110	10
3	*Lespedeza dahurica*	4	40	15
4	*Lespedeza dahurica*	5	30	15
5	*Lespedeza dahurica*	6	50	30
	Acer ginnala	3	120	15

Site: Caijiachuan forest farm **Forest type:** *Pinus tabulaeformis* plantation

Protected age: 60 a **Subplot area:** 1 m ×1 m **Mean coverage**: 27%

Investigator: Lin Hou **Date:** 2004.09.22

Subplot No.	Herb species	Individuals	Mean height (cm)	Coverage (%)
1	*Kengia serotina*	5	60	8
	Bothriochloa ischaemum	200(branches)	50	15
	Scutellaria baicalensis	3	50	8
2	*Kengia serotina*	5	50	8
	Bupleurum chinense	2	90	10
	Aster tataricus	1	50	3
3	*Bothriochloa ischaemum*	200(branches)	60	15
	Scutellaria baicalensis	4	40	5
	Anaphalis margaritacea	3	50	10
4	*Kengia serotina*	10	40	10
	Vicia cracca	1	80	5
	Artemisia gmelinii	2	50	5
5	*Bothriochloa ischaemum*	400(branches)	55	20
	Artemisia gmelinii	3	70	15

Site: Caijiachuan forest farm **Forest type:** *Pinus tabulaeformis* plantation

Protected age: 60 a **Plot area:** 20 m ×20 m **Canopy density**: 0.40

Altitude: 1130 m **Direction:** North **Gradient:** **16°** **Position:** Lower

Plot No. : 23 **Investigator:** Lin Hou **Date:** 2004.09.23

No.	Tree species	DBH/ground diameter（cm）	H (m)	Remark
1	*Quercus Liaotungensis*	4.5	6.4	
2	*Pinus tabulaeformis*	1.6	0.23	
3	*Pinus tabulaeformis*	1.5	0.3	
4	*Pinus tabulaeformis*	1.5	0.3	
5	*Pinus tabulaeformis*	18.5	25.5	
6	*Pinus tabulaeformis*	1.8	0.3	
7	*Pinus tabulaeformis*	1.6	0.25	
8	*Pinus tabulaeformis*	1.6	0.27	
9	*Pinus tabulaeformis*	11.5	17.0	
10	*Pinus tabulaeformis*	76.0	30.5	
11	*Pinus tabulaeformis*	11.6	20.4	
12	*Pinus tabulaeformis*	11.5	20.5	
13	*Pinus tabulaeformis*	1.9	0.3	
14	*Pinus tabulaeformis*	1.1	0.25	
15	*Pinus tabulaeformis*	1.9	0.3	
16	*Pinus tabulaeformis*	1.8	0.29	
17	*Populus davidiana*	3.8	4.2	
18	*Pinus tabulaeformis*	1.5	0.3	
19	*Pinus tabulaeformis*	1.6	0.29	
20	*Pinus tabulaeformis*	18.2	24.0	
21	*Pinus tabulaeformis*	11.8	23.0	
22	*Pinus tabulaeformis*	11.5	23.0	
23	*Pinus tabulaeformis*	11.4	23.0	
24	*Pinus tabulaeformis*	11.6	22.8	
25	*Pinus tabulaeformis*	1.4	0.21	
26	*Pinus tabulaeformis*	60.0	27.0	
27	*Pinus tabulaeformis*	3.5	1.8	
28	*Pinus tabulaeformis*	4.0	1.8	
29	*Pinus tabulaeformis*	11.0	23.5	
30	*Pinus tabulaeformis*	1.2	0.29	
31	*Pinus tabulaeformis*	1.9	0.3	
32	*Pinus tabulaeformis*	1.6	0.3	

33	*Pinus tabulaeformis*	17.8	26.5	
34	*Pinus tabulaeformis*	1.4	0.3	
35	*Pinus tabulaeformis*	1.6	0.3	
36	*Pinus tabulaeformis*	1.5	0.28	
37	*Pinus tabulaeformis*	19.5	24.4	
38	*Pinus tabulaeformis*	1.6	0.3	
39	*Pinus tabulaeformis*	1.4	0.3	
40	*Pinus tabulaeformis*	1.6	0.3	
41	*Syringa oblata*	2.8	3.6	
42	*Pinus tabulaeformis*	1.5	0.3	
43	*Pinus tabulaeformis*	15.5	27.8	
44	*Pinus tabulaeformis*	5.8	1.5	
45	*Pinus tabulaeformis*	5.6	1.8	
46	*Pinus tabulaeformis*	12.5	23.5	
47	*Pinus tabulaeformis*	13.0	23.5	
48	*Pinus tabulaeformis*	53.0	32.0	
49	*Pinus tabulaeformis*	5.5	1.6	
50	*Pinus tabulaeformis*	5.4	1.6	
51	*Pinus tabulaeformis*	5.0	1.5	
52	*Populus davidiana*	3.0	4.5	
53	*Pinus tabulaeformis*	5.0	1.5	
54	*Pinus tabulaeformis*	5.5	1.5	
55	*Pinus tabulaeformis*	2.5	0.3	
56	*Pinus tabulaeformis*	16.5	22.0	
57	*Pinus tabulaeformis*	2.6	0.2	
58	*Pinus tabulaeformis*	2.5	0.3	
59	*Pinus tabulaeformis*	14.5	25.0	
60	*Pinus tabulaeformis*	2.4	0.2	
61	*Pinus tabulaeformis*	2.6	0.3	
62	*Pinus tabulaeformis*	2.5	0.2	
63	*Pinus tabulaeformis*	18.8	23.8	

Site: Caijiachuan forest farm **Forest type:** *Pinus tabulaeformis* plantation

Protected age: 60 a **Subplot area:** 2 m ×2 m **Mean coverage**: 27%

Investigator: Lin Hou **Date:** 2004.09.24

Subplot No.	Shrub species	Individuals	Mean height (cm)	Coverage (%)
1	*Lespedeza dahurica*	5	45	20
	Rubus corchorifolius	1	120	10
2	*Lespedeza dahurica*	4	40	20
3	*Lespedeza dahurica*	3	60	25
4	*Lespedeza dahurica*	2	40	15
	Acer ginnala	4	90	15
5	*Lespedeza dahurica*	6	30	20
	Spiraea fritschiana	2	150	10

Site: Caijiachuan forest farm **Forest type:** *Pinus tabulaeformis* plantation

Protected age: 60 a **Subplot area:** 1 m ×1 m **Mean coverage**: 43%

Investigator: Lin Hou **Date:** 2004.09.24

Subplot No.	Herb species	Individuals	Mean height (cm)	Coverage (%)
1	*Bothriochloa ischaemum*	600(branches)	40	25
	Scutellaria baicalensis	1	60	5
	Artemisia gmelinii	4	45	10
2	*Kengia serotina*	8	55	10
	Viola selkirkii	1	15	5
	Artemisia gmelinii	2	60	8
3	*Bothriochloa ischaemum*	300(branches)	50	25
	Urena lobata	1	80	10
	Kengia serotina	5	60	8
4	*Bothriochloa ischaemum*	600(branches)	40	30
	Kengia serotina	10	40	15
	Viola chaerophylloides	1	20	5
5	*Bothriochloa ischaemum*	200(branches)	70	20
	Artemisia gmelinii	5	30	15
	Bupleurum chinense	3	60	15
	Neottianthe cucullata	1	20	5

Site: Caijiachuan forest farm **Forest type:** *Pinus tabulaeformis* plantation

Protected age: 75 a **Plot area:** 20 m ×20 m **Canopy density**: 0.30

Altitude: 1205 m **Direction:** Northeast **Gradient:** **10°** **Position:** Middle

Plot No. : 24 **Investigator:** Lin Hou **Date:** 2004.09.26

No.	Tree species	DBH/ground diameter（cm）	H (m)	Remark
1	*Pinus tabulaeformis*	19.6	35.8	
2	*Pinus tabulaeformis*	11.2	24.5	
3	*Pinus tabulaeformis*	11.5	25.4	
4	*Pinus tabulaeformis*	4.5	0.25	
5	*Pinus tabulaeformis*	4.2	0.3	
6	*Pinus tabulaeformis*	4.3	0.28	
7	*Populus davidiana*	3.8	4.2	
8	*Pinus tabulaeformis*	3.8	0.3	
9	*Pinus tabulaeformis*	3.9	0.28	
10	*Pinus tabulaeformis*	4.6	0.3	
11	*Pinus tabulaeformis*	4.5	0.27	
12	*Pinus tabulaeformis*	4.4	0.3	
13	*Pinus tabulaeformis*	5.2	1.8	
14	*Pinus tabulaeformis*	5.2	1.9	
15	*Pinus tabulaeformis*	19.6	35.8	
16	*Pinus tabulaeformis*	170.0	38.4	
17	*Pinus tabulaeformis*	4.5	1.4	
18	*Pinus tabulaeformis*	4.8	1.4	
19	*Pinus tabulaeformis*	4.6	0.28	
20	*Pinus tabulaeformis*	4.2	0.26	
21	*Pinus tabulaeformis*	4.4	0.29	
22	*Pinus tabulaeformis*	4.5	0.27	
23	*Toxicodendron vernicifluum*	3.5	4.6	
24	*Pinus tabulaeformis*	3.9	0.26	
25	*Pinus tabulaeformis*	4.1	0.28	
26	*Pinus tabulaeformis*	4.2	0.27	
27	*Pinus tabulaeformis*	3.8	0.3	
28	*Pinus tabulaeformis*	4.5	0.2	
29	*Pinus tabulaeformis*	4.4	0.2	
30	*Pinus tabulaeformis*	4.2	0.28	
31	*Pinus tabulaeformis*	4.2	0.29	
32	*Pinus tabulaeformis*	4.6	0.30	
33	*Pinus tabulaeformis*	4.8	0.28	
34	*Pinus tabulaeformis*	5.5	1.8	
35	*Pinus tabulaeformis*	5.2	1.6	
36	*Pinus tabulaeformis*	5.6	1.9	
37	*Pinus tabulaeformis*	11.8	33.6	

38	*Pinus tabulaeformis*	19.5	36.2	
39	*Pinus tabulaeformis*	165.0	38.5	
40	*Pinus tabulaeformis*	19.5	35.4	
41	*Pinus tabulaeformis*	5.8	1.7	
42	*Pinus tabulaeformis*	5.2	1.8	
43	*Pinus tabulaeformis*	5.5	1.8	
44	*Pinus tabulaeformis*	4.3	0.28	
45	*Pinus tabulaeformis*	4.8	0.3	
46	*Pinus tabulaeformis*	5.0	0.26	
47	*Pinus tabulaeformis*	4.3	0.3	
48	*Pinus tabulaeformis*	5.6	1.5	
49	*Quercus Liaotungensis*	1.9	3.2	
50	*Pinus tabulaeformis*	3.9	0.28	
51	*Pinus tabulaeformis*	4.8	0.3	
52	*Pinus tabulaeformis*	4.2	0.27	
53	*Pinus tabulaeformis*	5.8	1.6	
54	*Pinus tabulaeformis*	4.5	0.30	
55	*Pinus tabulaeformis*	4.4	0.25	
56	*Pinus tabulaeformis*	5.8	1.7	
57	*Pinus tabulaeformis*		0.30	
58	*Pinus tabulaeformis*	4.8	0.28	
59	*Pinus tabulaeformis*	4.6	0.18	
60	*Pinus tabulaeformis*	4.5	0.30	
61	*Pinus tabulaeformis*	5.2	0.30	
62	*Pinus tabulaeformis*	4.8	0.26	
63	*Pinus tabulaeformis*	4.8	0.28	
64	*Pinus tabulaeformis*	5.3	0.3	
65	*Syringa oblata*	2.8	3.6	
66	*Pinus tabulaeformis*	5.8	1.8	
67	*Pinus tabulaeformis*	5.8	1.8	
68	*Pinus tabulaeformis*	5.5	1.9	
69	*Pinus tabulaeformis*	11.5	31.2	
70	*Pinus tabulaeformis*	19.5	34.8	
71	*Pinus tabulaeformis*	8.8	28.4	
72	*Pinus tabulaeformis*	5.8	1.9	
73	*Pinus tabulaeformis*	5.8	1.9	
74	*Pinus tabulaeformis*	5.8	1.9	
75	*Pinus tabulaeformis*	11.4	31.5	
76	*Pinus tabulaeformis*	11.5	31.8	

77	*Pinus tabulaeformis*	11.4	31.4	
78	*Pinus tabulaeformis*	19.5	29.4	
79	*Pinus tabulaeformis*	11.5	25.6	
80	*Pinus tabulaeformis*	11.2	25.8	

Site: Caijiachuan forest farm **Forest type:** *Pinus tabulaeformis* plantation

Protected age: 75 a **Subplot area:** 2 m ×2 m **Mean coverage**: 39%

Investigator: Lin Hou **Date:** 2004.09.27

Subplot No.	Shrub species	Individuals	Mean height (cm)	Coverage (%)
1	*Acer ginnala*	2	120	15
	Clematis fruticosa	1	75	10
	Spiraea fritschiana	1	150	5
	Lespedeza dahurica	4	40	12
	Indigofera amblyantha	1	80	8
2	*Acer ginnala*	1	140	10
	Clematis fruticosa	1	70	10
	Lespedeza dahurica	3	50	10
	Rubus corchorifolius	1	120	5
3	*Acer ginnala*	2	90	10
	Clematis fruticosa	1	65	8
	Lespedeza dahurica	2	60	8
	Rubus corchorifolius	1	100	4
	Spiraea fritschiana	1	120	4
4	*Acer ginnala*	1	150	15
	Lespedeza dahurica	1	70	5
	Lonicera maccki	1	450	8
	Ostryopsis davidiana	1	120	5
5	*Acer ginnala*	2	80	10
	Clematis fruticosa	2	60	15
	Lonicera maccki	1	500	9
	Cotoneaster zbakelii	1	120	8

Site: Caijiachuan forest farm **Forest type:** *Pinus tabulaeformis* plantation

Protected age: 75 a **Subplot area:** 1 m ×1 m **Mean coverage**: 32%

Investigator: Lin Hou **Date:** 2004.09.27

Subplot No.	Herb species	Individuals	Mean height (cm)	Coverage (%)
1	*Bothriochloa ischaemum*	600(branches)	30	25
	Spodiopogon sibiricus	4	90	5
2	*Bothriochloa ischaemum*	300(branches)	50	20
	Kengia serotina	30	30	15
	Artemisia mongolica	2	60	5
3	*Bothriochloa ischaemum*	600(branches)	45	25
	Kengia serotina	30	35	15
4	*Bothriochloa ischaemum*	200(branches)	60	15
	Spodiopogon sibiricus	5	110	5
5	*Bothriochloa ischaemum*	200(branches)	70	15
	Kengia serotina	20	50	10
	Urena lobata	1	80	5

Site: Caijiachuan forest farm **Forest type:** *Pinus tabulaeformis* plantation

Protected age: 75 a **Plot area:** 20 m ×20 m **Canopy density**: 0.30

Altitude: 1200 m **Direction:** Northeast **Gradient:** 5° **Position:** Lower

Plot No. : 25 **Investigator:** Lin Hou **Date:** 2004.09.28

No.	Tree species	DBH/ground diameter（cm）	H (m)	Remark
1	*Syringa oblata*	2.8	3.4	
2	*Pinus tabulaeformis*	19.5	26.2	
3	*Pinus tabulaeformis*	2.4	0.19	
4	*Pinus tabulaeformis*	2.4	0.19	
5	*Pinus tabulaeformis*	4.5	1.9	
6	*Pinus tabulaeformis*	2.2	0.18	
7	*Pinus tabulaeformis*	2.2	0.18	
8	*Pinus tabulaeformis*	2.2	0.18	
9	*Pinus tabulaeformis*	2.2	0.14	
10	*Pinus tabulaeformis*	2.5	0.28	
11	*Pinus tabulaeformis*	4.3	1.8	
12	*Pinus tabulaeformis*	18.4	23.8	

13	*Pinus tabulaeformis*	4.4	1.8	
14	*Pinus tabulaeformis*	4.2	1.6	
15	*Populus davidiana*	3.6	5.2	
16	*Pinus tabulaeformis*	11.5	23.6	
17	*Pinus tabulaeformis*	85.0	34.5	
18	*Pinus tabulaeformis*	4.5	1.8	
19	*Pinus tabulaeformis*	4.5	1.6	
20	*Pinus tabulaeformis*	3.4	0.30	
21	*Pinus tabulaeformis*	2.6	0.15	
22	*Pinus tabulaeformis*	2.8	0.3	
23	*Pinus tabulaeformis*	18.4	26.8	
24	*Pinus tabulaeformis*	4.2	1.6	
25	*Pinus tabulaeformis*	2.4	0.27	
26	*Pinus tabulaeformis*	2.3	0.25	
27	*Pinus tabulaeformis*	1.9	0.14	
28	*Pinus tabulaeformis*	2.2	0.24	
29	*Pinus tabulaeformis*	1.6	0.16	
30	*Pinus tabulaeformis*	4.2	1.6	
31	*Pinus tabulaeformis*	18.5	26.2	
32	*Pinus tabulaeformis*	4.5	1.9	
33	*Quercus Liaotungensis*	2.6	4.1	
34	*Pinus tabulaeformis*	1.9	0.18	
35	*Pinus tabulaeformis*	2.2	0.28	
36	*Pinus tabulaeformis*	2.1	0.23	
37	*Pinus tabulaeformis*	4.8	1.8	
38	*Pinus tabulaeformis*	4.6	1.5	
39	*Pinus tabulaeformis*	11.6	24.5	
40	*Pinus tabulaeformis*	65	42	
41	*Pinus tabulaeformis*	11.4	24.6	
42	*Pinus tabulaeformis*	3.2	1.6	
43	*Pinus tabulaeformis*	3.0	1.5	
44	*Pinus tabulaeformis*	2.8	0.29	
45	*Pinus tabulaeformis*	2.5	0.24	
46	*Pinus tabulaeformis*	4.0	1.6	
47	*Pinus tabulaeformis*	19.8	27.6	
48	*Pinus tabulaeformis*	4.8	1.8	
49	*Pinus tabulaeformis*	2.6	0.25	
50	*Pinus tabulaeformis*	2.5	0.26	
51	*Pinus tabulaeformis*	2.4	0.28	

52	*Pinus tabulaeformis*	4.9	1.8	
53	*Pinus tabulaeformis*	11.8	23.8	
54	*Pinus tabulaeformis*	54.0	33.2	
55	*Pinus tabulaeformis*	11.9	23.6	
56	*Pinus tabulaeformis*	2.8	1.8	
57	*Pinus tabulaeformis*	3.4	1.8	
58	*Pinus tabulaeformis*	2.5	0.28	
59	*Pinus tabulaeformis*	2.5	0.28	
60	*Quercus Liaotungensis*	3	5.4	
61	*Pinus tabulaeformis*	2.5	0.30	
62	*Pinus tabulaeformis*	17.5	26.5	
63	*Pinus tabulaeformis*	2.2	0.3	
64	*Pinus tabulaeformis*	2.4	0.24	
65	*Pinus tabulaeformis*	11.5	23.3	
66	*Pinus tabulaeformis*	36.0	30.0	
67	*Pinus tabulaeformis*	3.6	1.9	
68	*Pinus tabulaeformis*	3.8	1.8	
69	*Pinus tabulaeformis*	1.8	0.2	
70	*Pinus tabulaeformis*	1.8	0.16	
71	*Pinus tabulaeformis*	1.8	0.23	
72	*Pinus tabulaeformis*	16.6	26.8	

Site: Caijiachuan forest farm **Forest type:** *Pinus tabulaeformis* plantation

Protected age: 75 a **Subplot area:** 2 m ×2 m **Mean coverage**: 40%

Investigator: Lin Hou **Date:** 2004.09.29

Subplot No.	Shrub species	Individuals	Mean height (cm)	Coverage (%)
1	*Acer ginnala*	1	130	8
	Lespedeza dahurica	3	55	10
	Indigofera amblyantha	1	90	9
2	*Acer ginnala*	3	90	15
	Clematis fruticosa	3	55	15
	Lespedeza dahurica	4	45	12
	Rubus corchorifolius	1	150	5
	Spiraea fritschiana	1	180	8
3	*Acer ginnala*	1	100	9
	Clematis fruticosa	1	80	12

	Lonicera maccki	1	400	6
	Cotoneaster zbakelii	1	150	10
4	*Acer ginnala*	2	75	10
	Clematis fruticosa	1	60	10
	Lespedeza dahurica	3	50	10
	Rubus corchorifolius	1	120	4
	Spiraea fritschiana	1	150	5
5	*Acer ginnala*	3	60	12
	Clematis fruticosa	1	70	10
	Lespedeza dahurica	2	60	8
	Rubus corchorifolius	1	150	5
	Ostryopsis davidiana	1	150	8

Site: Caijiachuan forest farm **Forest type:** *Pinus tabulaeformis* plantation

Protected age: 75 a **Subplot area:** 1 m ×1 m **Mean coverage**: 37%

Investigator: Lin Hou **Date:** 2004.09.29

Subplot No.	Herb species	Individuals	Mean height (cm)	Coverage (%)
1	*Bothriochloa ischaemum*	200(branches)	50	20
	Kengia serotina	30	40	15
	Artemisia mongolica	3	50	6
2	*Bothriochloa ischaemum*	600(branches)	40	25
	Kengia serotina	20	45	10
	Aster tataricus	1	45	3
3	*Bothriochloa ischaemum*	400(branches)	55	20
	Spodiopogon sibiricus	3	95	5
	Sanguisorba officinalis	1	60	5
4	*Bothriochloa ischaemum*	600(branches)	45	25
	Kengia serotina	30	40	15
5	*Bothriochloa ischaemum*	600(branches)	35	25
	Kengia serotina	15	50	10

Site: Caijiachuan forest farm **Forest type:** *Pinus tabulaeformis* plantation

Protected age: 75 a **Plot area:** 20 m ×20 m **Canopy density**: 0.30

Altitude: 1185 m **Direction:** **Gradient:** ° **Position:** Mesa

Plot No. : 26 **Investigator:** Lin Hou **Date:** 2004.09.30

No.	Tree species	DBH/ground diameter（cm）	H (m)	Remark
1	*Pinus tabulaeformis*	45.6		
2	*Pinus tabulaeformis*	10.2		
3	*Pinus tabulaeformis*	5.5		
4	*Syringa oblata*	3.8		
5	*Pinus tabulaeformis*	4.0		
6	*Pinus tabulaeformis*	2.8		
7	*Pinus tabulaeformis*	5.2		
8	*Pinus tabulaeformis*	5.6		
9	*Pinus tabulaeformis*	38.3		
10	*Pinus tabulaeformis*	4.1		
11	*Pinus tabulaeformis*	3.9		
12	*Pinus tabulaeformis*	6.7		
13	*Populus davidiana*	3.6		
14	*Pinus tabulaeformis*	10.9		
15	*Pinus tabulaeformis*	8.2		
16	*Pinus tabulaeformis*	0.8		
17	*Pinus tabulaeformis*	4.6		
18	*Pinus tabulaeformis*	5.8		
19	*Pinus tabulaeformis*	30.6		
20	*Pinus tabulaeformis*	7.8		
21	*Pinus tabulaeformis*	4.2		
22	*Pinus tabulaeformis*	2.9		
23	*Pinus tabulaeformis*	10.5		
24	*Pinus tabulaeformis*	10.1		
25	*Pinus tabulaeformis*	4.1		
26	*Pinus tabulaeformis*	14.0		
27	*Pinus tabulaeformis*	13.0		
28	*Pinus tabulaeformis*	16.7		
29	*Pinus tabulaeformis*	11.6		
30	*Quercus Liaotungensis*	4		
31	*Pinus tabulaeformis*	6.8		
32	*Pinus tabulaeformis*	1.0		

33	*Pinus tabulaeformis*	22.5		
34	*Pinus tabulaeformis*	23.9		
35	*Pinus tabulaeformis*	26.8		
36	*Pinus tabulaeformis*	25.7		
37	*Pinus tabulaeformis*	12.4		
38	*Pinus tabulaeformis*	13.5		
39	*Pinus tabulaeformis*	4.0		
40	*Pinus tabulaeformis*	17.6		
41	*Pinus tabulaeformis*	5.6		
42	*Pinus tabulaeformis*	10.6		
43	*Pinus tabulaeformis*	7.3		
44	*Pinus tabulaeformis*	13.0		
45	*Pinus tabulaeformis*	6.5		
46	*Pinus tabulaeformis*	7.6		
47	*Pinus tabulaeformis*	8.0		
48	*Pinus tabulaeformis*	16.8		
49	*Pinus tabulaeformis*	6.1		
50	*Pinus tabulaeformis*	8.6		
51	*Pinus tabulaeformis*	45.4		
52	*Pinus tabulaeformis*	20.5		
53	*Pinus tabulaeformis*	16.2		
54	*Pinus tabulaeformis*	32.5		
55	*Pinus tabulaeformis*	10.5		
56	*Pinus tabulaeformis*	14.4		
57	*Pinus tabulaeformis*	10.60		
58	*Pinus tabulaeformis*	4.60		
59	*Pinus tabulaeformis*	4.60		

Site: Caijiachuan forest farm **Forest type:** *Pinus tabulaeformis* plantation

Protected age: 75 a **Subplot area:** 2 m ×2 m **Mean coverage**: 41%

Investigator: Lin Hou **Date:** 2004.10.03

Subplot No.	Shrub species	Individuals	Mean height (cm)	Coverage (%)
1	*Acer ginnala*	1	150	10
	Lespedeza dahurica	4	50	15
	Cotoneaster zbakelii	1	170	12
2	*Acer ginnala*	1	170	12
	Clematis fruticosa	2	65	15

	Rubus corchorifolius	1	120	3
	Lespedeza dahurica	5	40	13
	Spiraea fritschiana	1	140	5
3	*Acer ginnala*	1	160	11
	Clematis fruticosa	3	55	13
	Rubus corchorifolius	1	150	5
	Ostryopsis davidiana	1	200	10
4	*Acer ginnala*	1	150	10
	Clematis fruticosa	4	50	15
	Lespedeza dahurica	5	55	15
	Lonicera maccki	1	550	10
5	*Acer ginnala*	2	85	10
	Lespedeza dahurica	4	45	15
	Rubus corchorifolius	1	120	3

Site: Caijiachuan forest farm **Forest type:** *Pinus tabulaeformis* plantation

Protected age: 75 a **Subplot area:** 1 m ×1 m **Mean coverage**: 29%

Investigator: Lin Hou **Date:** 2004.10.03

Subplot No.	Herb species	Individuals	Mean height (cm)	Coverage (%)
1	*Bothriochloa ischaemum*	600(branches)	40	25
	Spodiopogon sibiricus	4	100	5
	Thalictrum prezewalskii	1	90	5
2	*Bothriochloa ischaemum*	200(branches)	60	15
	Kengia serotina	40	35	15
	Viola chaerophylloides	1	15	3
3	*Bothriochloa ischaemum*	200(branches)	50	15
	Kengia serotina	40	30	15
4	*Bothriochloa ischaemum*	200(branches)	55	15
	Spodiopogon sibiricus	4	90	5
5	*Kengia serotina*	40	35	15
	Artemisia mongolica	1	80	4

Site: Caijiachuan forest farm **Forest type:** *Pinus tabulaeformis* plantation

Protected age: 75 a **Plot area:** 20 m ×20 m **Canopy density**: 0.40

Altitude: 1135 m **Direction:** North **Gradient:** 18° **Position:** Middle

Plot No. : 27 **Investigator:** Lin Hou **Date:** 2004.10.04

No.	Tree species	DBH/ground diameter（cm）	H (m)	Remark
1	*Pinus tabulaeformis*	5.2	0.25	
2	*Pinus tabulaeformis*	5.3	0.2	
3	*Pinus tabulaeformis*	5.4	0.3	
4	*Syringa oblata*	2.8	3.6	
5	*Pinus tabulaeformis*	5.9	1.8	
6	*Pinus tabulaeformis*	5.9	1.6	
7	*Pinus tabulaeformis*	5.9	1.8	
8	*Pinus tabulaeformis*	5.4	0.25	
9	*Pinus tabulaeformis*	5.5	0.25	
10	*Pinus tabulaeformis*	5.8	1.8	
11	*Pinus tabulaeformis*	5.8	1.8	
12	*Pinus tabulaeformis*	19.6	32.9	
13	*Populus davidiana*	3.2	4.0	
14	*Pinus tabulaeformis*	5.4	0.25	
15	*Pinus tabulaeformis*	5.3	0.3	
16	*Pinus tabulaeformis*	5.8	1.8	
17	*Pinus tabulaeformis*	5.8	1.8	
18	*Pinus tabulaeformis*	5.9	1.8	
19	*Pinus tabulaeformis*	11.8	30.6	
20	*Pinus tabulaeformis*	11.8	30.8	
21	*Pinus tabulaeformis*	11.8	30.4	
22	*Pinus tabulaeformis*	156.0	38.6	
23	*Pinus tabulaeformis*	11.9	30.6	
24	*Pinus tabulaeformis*	11.9	30.8	
25	*Pinus tabulaeformis*	5.9	1.9	
26	*Pinus tabulaeformis*	5.8	1.8	
27	*Pinus tabulaeformis*	19.5	32.8	
28	*Pinus tabulaeformis*	5.4	0.18	
29	*Pinus tabulaeformis*	5.5	0.2	
30	*Quercus Liaotungensis*	3.8	5.1	
31	*Pinus tabulaeformis*	5.4	0.15	
32	*Pinus tabulaeformis*	5.2	0.28	
33	*Pinus tabulaeformis*	5.9	1.8	
34	*Pinus tabulaeformis*	5.8	1.8	
35	*Pinus tabulaeformis*	5.8	1.8	
36	*Pinus tabulaeformis*	19.5	32.5	
37	*Pinus tabulaeformis*	5.4	0.25	

38	*Pinus tabulaeformis*	5.4	0.25	
39	*Pinus tabulaeformis*	5.3	0.25	
40	*Pinus tabulaeformis*	11.8	31.6	
41	*Pinus tabulaeformis*	11.8	31.5	
42	*Pinus tabulaeformis*	5.5	0.28	
43	*Pinus tabulaeformis*	5.4	0.25	
44	*Pinus tabulaeformis*	5.2	0.28	
45	*Pinus tabulaeformis*	19.6	32.4	
46	*Pinus tabulaeformis*	5.4	0.29	
47	*Pinus tabulaeformis*	5.5	0.29	
48	*Pinus tabulaeformis*	5.5	0.29	
49	*Pinus tabulaeformis*	11.8	30.2	
50	*Pinus tabulaeformis*	11.9	30.4	
51	*Pinus tabulaeformis*	5.7	1.8	
52	*Pinus tabulaeformis*	5.9	1.7	
53	*Pinus tabulaeformis*	5.9	1.9	
54	*Pinus tabulaeformis*	5.6	0.28	
55	*Pinus tabulaeformis*	5.5	0.29	
56	*Pinus tabulaeformis*	19.5	33.6	
57	*Pinus tabulaeformis*	5.3	0.25	
58	*Pinus tabulaeformis*	5.2	0.25	
59	*Pinus tabulaeformis*	11.5	31.5	

Site: Caijiachuan forest farm **Forest type:** *Pinus tabulaeformis* plantation

Protected age: 75 a **Subplot area:** 2 m ×2 m **Mean coverage**: 36%

Investigator: Lin Hou **Date:** 2004.10.05

Subplot No.	Shrub species	Individuals	Mean height (cm)	Coverage (%)
1	*Acer ginnala*	3	90	15
	Clematis fruticosa	2	60	10
	Rubus corchorifolius	1	150	5
	Indigofera amblyantha	1	150	12
2	*Acer ginnala*	2	100	15
	Clematis fruticosa	1	70	9
	Lespedeza dahurica	5	40	12
	Rubus corchorifolius	1	160	6
3	*Acer ginnala*	1	90	8

	Clematis fruticosa	1	75	10
	Lespedeza dahurica	3	50	8
	Ostryopsis davidiana	1	150	8
4	*Acer ginnala*	3	60	8
	Clematis fruticosa	1	70	8
	Lespedeza dahurica	2	55	6
	Spiraea fritschiana	1	150	5
	Lonicera maccki	1	500	8
5	*Acer ginnala*	2	80	8
	Clematis fruticosa	2	60	10
	Rubus corchorifolius	1	150	5
	Spiraea fritschiana	1	120	4

Site: Caijiachuan forest farm **Forest type:** *Pinus tabulaeformis* plantation

Protected age: 75 a **Subplot area:** 1 m ×1 m **Mean coverage**: 35%

Investigator: Lin Hou **Date:** 2004.10.05

Subplot No.	Herb species	Individuals	Mean height (cm)	Coverage (%)
1	*Bothriochloa ischaemum*	300(branches)	45	20
	Kengia serotina	30	40	15
2	*Bothriochloa ischaemum*	600(branches)	30	25
	Anaphalis margaritacea	3	40	8
3	*Bothriochloa ischaemum*	600(branches)	35	25
	Kengia serotina	20	50	10
4	*Bothriochloa ischaemum*	600(branches)	45	25
	Anaphalis margaritacea	2	45	6
	Vicia unijuga	1	70	3
5	*Bothriochloa ischaemum*	600(branches)	40	25
	Kengia serotina	20	40	10
	Viola selkirkii	1	14	3
