# Supplementary material for: How long should the fully hillside-closed forest protection be implemented on the Loess Plateau, Shaanxi, China?
Source: PeerJ. 2017 Sep 4;5:e3764. doi: 10.7717/peerj.3764 (PMC5588796; doi:10.7717/peerj.3764)
Supplement: Supplemental Information 2 [file peerj-05-3764-s002.pdf]

Chemical analysis

Protected age	Plot NO.	SOC (gkg^{-1})	NH_4^+-N ($mgkg^{-1}$)	NO_3^--N ($mgkg^{-1}$)	Available P ($mgkg^{-1}$)	Available K ($mgkg^{-1}$)
16	1	24.94	2.72	23.42	3.12	176.84
16	2	26.08	3.01	24.09	2.38	180.25
16	3	24.35	2.88	20.8	3.05	184.01
16	4	25.98	2.84	21.87	4.04	183.86
16	5	26.35	2.85	21.82	3.06	183.24
30	6	17.98	3.01	23.54	4.08	178.24
30	7	18.65	3.44	25.12	3.42	174.33
30	8	18.74	3.51	24.86	3.14	172.56
30	9	18.87	3.14	23.93	3.66	177.77
30	10	17.42	3.56	24.78	3.51	174.49
30	11	19.52	3.26	21.95	3.61	173.69
45	12	22.89	2.28	19.32	3.65	160.20
45	13	24.01	2.12	20.01	3.24	164.30
45	14	25.12	2.56	17.86	4.02	161.90
45	15	23.31	2.45	18.42	3.56	163.80
45	16	22.87	2.61	18.24	3.98	165.10
45	17	23.52	2.25	17.94	4.26	160.90
45	18	22.94	2.03	18.12	3.63	162.80
45	19	24.22	2.10	18.57	3.90	161.00
60	20	28.45	2.45	18.86	3.98	162.32
60	21	31.06	2.75	22.34	4.12	165.87
60	22	28.79	2.88	20.94	4.68	166.05
60	23	30.7	2.52	18.9	4.18	165.32
75	24	36.84	2.26	20.24	4.21	188.98
75	25	33.56	2.64	18.68	4.94	192.14
75	26	32.98	2.32	21.12	4.86	193.26
75	27	33.54	2.38	19.32	4.83	187.34
